# Simultaneous membrane and RNA binding by tick-borne encephalitis virus capsid protein

**DOI:** 10.1371/journal.ppat.1011125

**Published:** 2023-02-14

**Authors:** Lauri Ilmari Aurelius Pulkkinen, Sarah Victoria Barrass, Marie Lindgren, Hudson Pace, Anna K. Överby, Maria Anastasina, Marta Bally, Richard Lundmark, Sarah Jane Butcher

**Affiliations:** 1 Faculty of Biological and Environmental Sciences, Molecular and Integrative Bioscience Research Programme, University of Helsinki, Helsinki, Finland; 2 Helsinki Institute of Life Sciences-Institute of Biotechnology, University of Helsinki, Helsinki, Finland; 3 Department of Clinical Microbiology, Faculty of Medicine, Umeå University, Umeå, Sweden; 4 The Laboratory for Molecular Infection Medicine Sweden (MIMS), Umeå University, Umeå, Sweden; 5 Wallenberg Centre for Molecular Medicine, Umeå University, Umeå, Sweden; 6 Department of Integrative Medical Biology, Faculty of Medicine, Umeå University, Umeå, Sweden; National Institute of Allergy and Infectious Diseases, UNITED STATES

## Abstract

Tick-borne encephalitis virus is an enveloped, pathogenic, RNA virus in the family *Flaviviridae*, genus *Flavivirus*. Viral particles are formed when the nucleocapsid, consisting of an RNA genome and multiple copies of the capsid protein, buds through the endoplasmic reticulum membrane and acquires the viral envelope and the associated proteins. The coordination of the nucleocapsid components to the sites of assembly and budding are poorly understood. Here, we investigate the interactions of the wild-type and truncated capsid proteins with membranes with biophysical methods and model membrane systems. We show that capsid protein initially binds membranes via electrostatic interactions with negatively-charged lipids, which is followed by membrane insertion. Additionally, we show that membrane-bound capsid protein can recruit viral genomic RNA. We confirm the biological relevance of the biophysical findings by using mass spectrometry to show that purified virions contain negatively-charged lipids. Our results suggest that nucleocapsid assembly is coordinated by negatively-charged membrane patches on the endoplasmic reticulum and that the capsid protein mediates direct contacts between the nucleocapsid and the membrane.

## Author summary

Tick-borne encephalitis virus (TBEV) causes a life-threatening neurological disease. The incidence of the disease is increasing despite the availability of efficient vaccines. The virus particles contain a nucleocapsid consisting of the viral RNA genome and multiple copies of the capsid protein. The nucleocapsid is surrounded by an envelope derived from host membranes and two viral surface protein species are embedded into it. The assembly of TBEV virions is poorly understood despite its medical significance. Here, we have used a large combination of biophysical methods to study the interactions of the capsid protein with artificial lipid membranes. We show that the capsid protein requires negatively-charged lipids for membrane binding and that the capsid protein is capable of inserting into the membranes in the presence of such lipids. Once membrane-bound, the capsid protein is able to recruit TBEV genomic RNA onto the membrane. Our results suggest that the initial events of TBEV assembly are mediated by the specific binding of the capsid protein to negatively-charged lipids, allowing for the recruitment of the genome. This is further supported by our characterization of the lipid composition of purified TBEV particles, which shows that the TBEV envelope contains negatively-charged lipids.

## Introduction

Tick-borne encephalitis virus (TBEV) is a viral pathogen in the family *Flaviviridae*, genus *Flavivirus* [1,2]. TBEV is mainly spread by ticks, and causes life-threatening neurological symptoms with human fatality rates as high as 40% depending on the virus subtype [3,4]. Although an effective vaccine exists, the frequency of TBEV infections has increased dramatically in recent decades [3,4]. Furthermore, there are no antivirals available, and the treatment of patients is limited to symptomatic care [3,4].

The TBEV virion has a typical flavivirus structure [5,6]. The surface of the virion is made up of two protein species, E and M that are embedded in the virion envelope in an icosahedrally-symmetric lattice [5,6]. The nucleocapsid (NC) consists of a positive-strand RNA genome of about 11 kb and multiple copies of the capsid (C) protein. However, the structure of the NC inside the particle and its assembly both remain poorly understood [5–7]. The NC assembles at the endoplasmic reticulum (ER) and buds into the ER lumen acquiring both the lipid envelope and the envelope proteins during this process [1,7]. This yields immature, non-infectious particles that need to undergo both pH-mediated conformational changes as well as proteolytic cleavage to mature [8]. In addition to the NC itself, assembly and budding involve at least E, the M protein precursor (prM), the viral non-structural (NS) proteins NS2A, NS2B-NS3 and multiple host factors such as SPCS1, nucleolin, DDX56, and components of the endosomal sorting complex required for transport (ESCRT) [9,10,11–18,19–24]. Currently, it is not clear if the NC assembles before or concurrently with membrane budding, but since isolated NCs have not been observed in cells, the processes are likely interlinked [25–28]. The central role of the C protein is to recruit genomic RNA to the ER budding site either by interacting with the transmembrane domains of E and prM, host proteins, viral NS proteins, or specific ER lipids [7]. However, the regulation of these interactions is not understood.

The C protein is translated as part of the single, ER-embedded TBEV polyprotein. It initially has a C-terminal membrane-spanning anchor of 20 residues, which is cleaved by the viral NS2B-NS3 protease [29]. The mature form of TBEV C protein consists of 96 residues and it has a similar fold as the C proteins of other flaviviruses (S1 Fig) [30–34]. The TBEV C protein is mainly a dimer both in solution, and within the virion, although higher-order oligomerisation has been suggested to occur [34–37]. The C protein contains a flexible N-terminus (17 aa) and four α-helices (α_1–_α_4_), with dimerization occurring via antiparallel interactions of the α_2_ and α_4_ helices [7,30–34,37,38]. The α_4_ helix contains multiple positively-charged residues, thought to form a charged surface responsible for nucleic acid binding [32,34,35,39]. The other side of the dimer (the α_2_ helix or the N-terminus) may be important for nucleic acid or membrane binding, although this facet of C protein function remains under-studied [32,40–42].

As the C protein has membrane affinity, and the assembly process occurs at the ER, it is likely that specific ER lipids are involved in the process. Budding involves generating extreme membrane curvature which is only possible in the presence of suitable lipids. Flavivirus infection leads to a significant increase in curvature-promoting lipids in the host, and increases the curvature of the ER membrane [43–50]. Previous work has mainly concentrated on fluorescence microscopy-based characterization of C protein localization in infected cells, experiments conducted with C-derived peptides, and indirect characterization of membrane insertion [40,41,51–53]. Here, we have probed the role of lipids in the NC assembly by using recombinant TBEV C and various model lipid systems to biophysically characterize the interaction of the C protein with membranes. We show that the C protein initially binds to lipids with negatively-charged head groups via electrostatic interactions which is then followed by membrane insertion. We confirm the physiological relevance of the experimental system by showing that the C protein can recruit genomic RNA whilst membrane-bound in the presence or absence of the 17 N-terminal residues. Furthermore, we characterise the TBEV lipidome with ultra-high performance liquid chromatography-quadrupole time-of-flight mass spectrometry (UHPLC-QTOF-MS) and show that the TBEV virion contain lipids with negatively-charged head groups. Our results indicate that the C protein may be recruited to the ER via positively charged lipid head groups, helping to nucleate the assembly of the NC and acquisition of the membrane through budding into the ER.

## Results

### Purified C protein binds to lipids with negatively-charged head groups

The homology model of the full-length C protein from the Kuutsalo-14 strain was predicted using the I-TASSER server by using the structure of the truncated C protein (PDB: 7YWQ) as a template (S1B Fig) [34,54–57]. The C-score of the homology model was -0.69 (on a scale of -5 to 2), indicating high confidence structure prediction [54–56]. The homology model had an overall similar fold to the truncated C protein, with the N-terminus forming an additional α helix (S1B Fig). Electrostatic surface potential calculation showed that in addition to the positively-charged patch on the α_4_-α_4_ interface, the N-terminus is also positively charged (S1C Fig).

**The homology model was used to guide C protein mutagenesis (S1B Fig) and both the full-length C and the truncated C (**C_**18–93**_, containing **residues 18–93) were expressed in** *E*.*coli*. After immobilized metal affinity chromatography and ion-exchange chromatography, both proteins eluted as pure dimers in size-exclusion chromatography (SEC) confirmed by SDS-PAGE and immunoblotting (S2 Fig). The A_260_/A_230_ ratios of the purified proteins were 0.61±0.02 and 0.55±0.3 for C and C_**18–93**_ respectively indicating no significant nucleic acid contamination (average of three technical replicates) [58].

**Fig 1:**
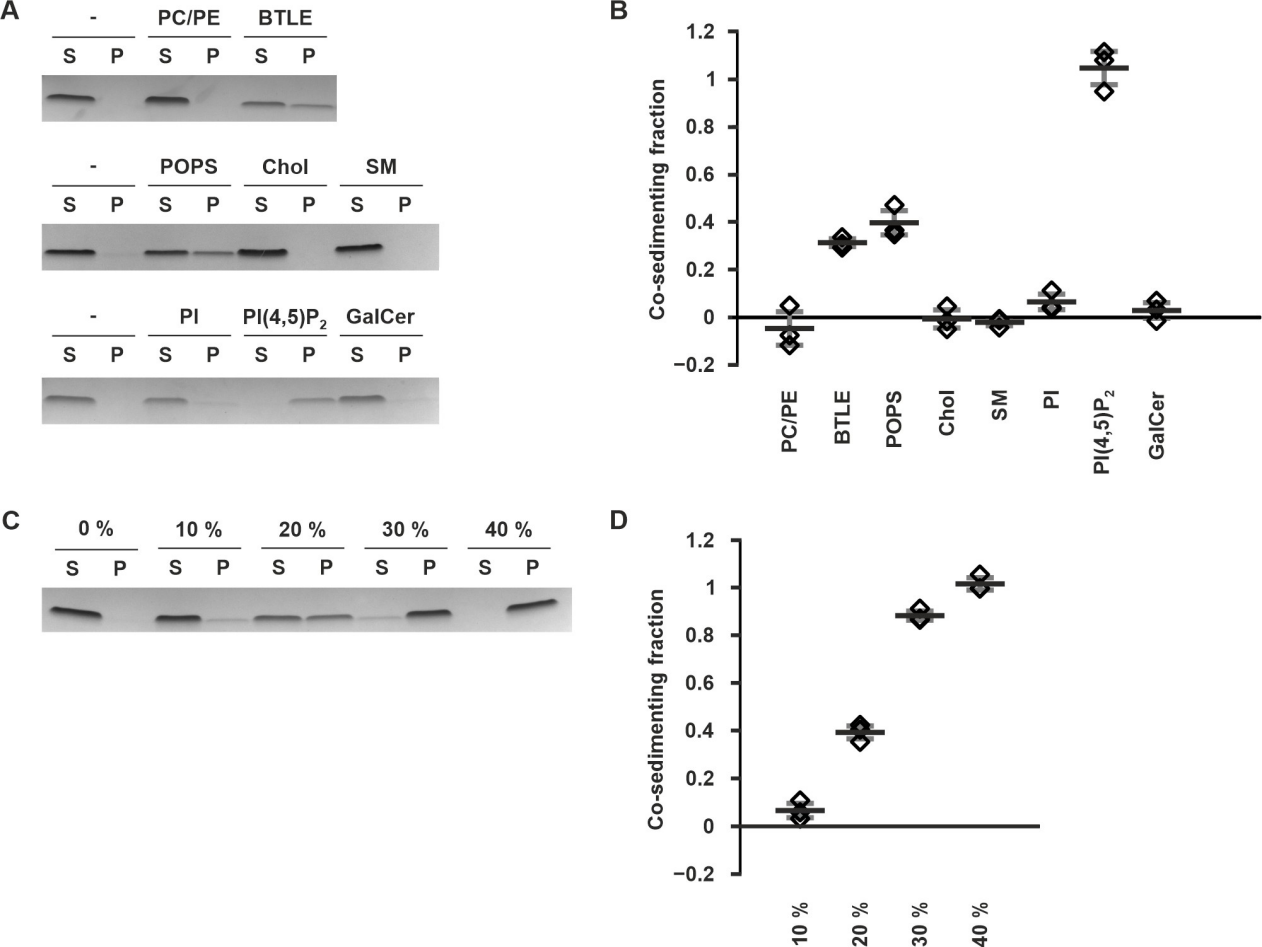
Full-length C protein binds preferentially to negatively-charged lipids. **A, B** Full-length C protein co-sedimentation with liposomes of different compositions. (**A**) shows representative SDS-PAGE bands with S indicating the supernatant and P the pellet. (**B**) shows densitometric quantitation of the co-sedimenting fraction from the pellet. PC/PE refers to pure DOPC/DOPE liposomes, BTLE to pure BTLE liposomes, a dash to the no liposome control and the other labels to DOPC/DOPE liposomes supplemented POPS, Chol, SM, PI, PI(4,5)P_2_ or GalCer. **C, D** Full-length C protein co-sedimentation with liposomes containing varying amounts of POPS. (**C**) shows representative SDS-PAGE bands where S indicates the supernatant and P the pellet with the POPS percentage indicated above the lanes. (**D**) shows densitometric quantitation of the co-sedimenting fraction from the pellet with the w/w percentage of POPS in the liposomes is indicated on the Y axis. Data information: In (**B**) and (**D**), data are shown as the averages of three replicates with the error bars representing the standard deviation (s.d.). Individual measurements are shown as diamonds. Data are normalized against no liposome (**B**) or 0% POPS control (**D**).

To determine if the full-length C protein bound to membranes and if lipid binding was dependent on the type of lipid head groups, we used liposome co-sedimentation. First, we used liposomes containing either brain total lipid extract (BTLE), or a mixture of the uncharged lipids 1,2-dioleoyl-sn-glycero-3-phosphocholine (DOPC) and 1,2-dioleoyl-sn-glycero-3-phosphoethanolamine (DOPE). Full-length C protein was found to bind and co-sediment with BTLE liposomes, but not with DOPC-DOPE (Fig 1A and 1B). Next, we supplemented the DOPC-DOPE liposomes with individual glycerophospholipids, sphingolipids or cholesterol (Chol) representing different specific lipid properties. Incorporation of sphingomyelin (SM), phosphatidylinositol (PI), or galactocerebrosides (GalCer) showed that lipids with uncharged or glycosylated head groups did not markedly increase full-length C protein binding, neither did Chol. However, clear binding was detected after incorporation of the negatively-charged 1-palmitoyl-2-oleoyl-sn-glycero-3-phospho-L-serine (POPS) or the highly negatively-charged L-α-phosphatidylinositol-4,5-bisphosphate (PI(4,5)P_2_) (Fig 1A and 1B). This indicated that full-length C bound to negatively-charged membranes without a preference for a specific head group. Furthermore, PI(4,5)P_2_ that is 3 times more charged than POPS resulted in increased binding (Fig 1A and 1B). To further confirm the role of charge, DOPC-DOPE liposomes were supplemented with increasing concentrations of POPS, increasing the negative charge of the liposomes proportionally, and co-sedimentation was then assessed. The fraction of co-sedimenting full-length C protein increased with increasing POPS concentrations, with all protein being bound at 40% POPS (Fig 1C and 1D). Thus, 40% POPS was used for further experiments.

We confirmed the results of the co-sedimentation with liposome flotation assays. Full-length C protein co-migrated with liposomes containing 40% POPS. The majority of the C protein was detected in the fraction containing the fluorescently-labelled lipids based on SDS-PAGE analysis and fluorescence measurements (S3 Fig). Contrastingly, in experiments with liposomes containing no negatively-charged lipids, the C protein did not co-migrate and the majority of the protein was detected in the fractions without liposomes (S3 Fig). Similar results were obtained in control experiments without liposomes (S3 Fig). This confirmed that C protein binds liposomes with charged lipid headgroups, rather than precipitating in the co-sedimentation assay.

### Initial C protein recruitment to the membrane is of an electrostatic nature

**Fig 2:**
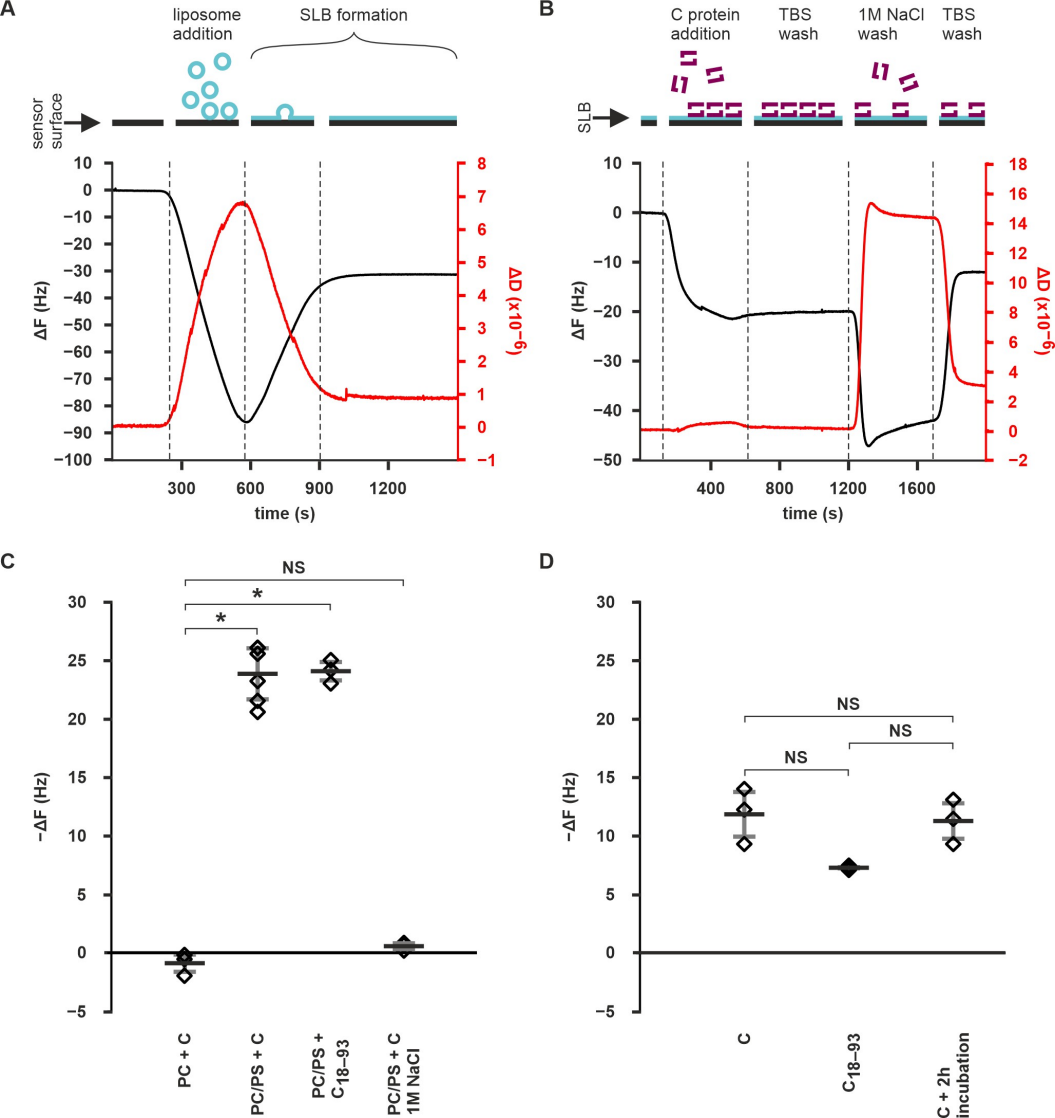
C protein binds to SLBs with negatively-charged lipids in QCM-D measurements. **A** Representative QCM-D curves from POPC/POPS SLB generation. The ΔF and ΔD have been zeroed to prelipid injection equilibrium values. **B** Representative QCM-D curves from a C protein binding experiment on POPC/POPS SLB. The ΔF and ΔD have been zeroed to equilibrium values after SLB formation. The large reversible changes in ΔF and ΔD after 1 M NaCl injection indicate a typical “buffer shift”, as the ΔF and ΔD values are sensitive to buffer viscosity [60]. **C** Quantitation of the final −ΔF values upon C protein and C_18–93_ injection on SLBs composed of pure POPC (PC), or POPC/POPS mixture (PC/PS) either in TBS or in 1 M NaCl-containing buffer. **D** Quantitation of the final −ΔF values on SLBs with prebound C or C_**18–93**_ after 1 M NaCl wash either right after equilibration, or after 2h incubation. Data information. Data in **C** and **D** are shown as the mean of 6 (C binding in TBS on POPC/POPS) or 3 (all others) replicates with the s.d. represented by the error bars. Individual measurements are shown as diamonds. Statistical significance (p <0.05) is indicated by an asterisk and non-significant differences with NS. See main text for the p-values.

To further characterise the binding of C protein to membranes, supported lipid bilayers (SLBs) on a quartz crystal microbalance with dissipation monitoring (QCM-D) measurements were used. SLBs were generated either from pure 1-palmitoyl-2-oleoyl-sn-glycero-3-phosphocholine (POPC) or a mixture of POPC and POPS (60:40% w/w) (Fig 2A). QCM-D measures the change in frequency (ΔF) of an oscillating quartz crystal which is related to the mass adsorbed to its surface. It also monitors the energy dissipation (ΔD) representing how rigid the adsorbed material is, a high dissipation corresponding to a more viscoelastic (soft) film. During formation of SLBs the adsorption of liposomes and subsequent fusion into a two-dimensional rigid SLB can be scored using these parameters (Fig 2A) [59]. After the formation of the SLB and the equilibration of the system with Tris-buffered saline (TBS), the ΔF and ΔD values were zeroed, and the C protein was injected. The C protein injection caused the ΔF to decrease to −23.9 Hz on average (s.d. 2.2 Hz) in POPC/POPS experiments indicating a clear binding of additional mass on to the SLB (Fig 2B and 2C). However, repeating the experiment in the presence of pure POPC SLBs, resulted in a significantly smaller ΔF after C protein injection (on average 0.9 Hz, s.d. 0.88 Hz) indicating no mass binding (Figs 2C and S4). The ΔF difference between protein binding to the POPC/POPS and POPC SLBs was statistically significant (p-value 6.94×10^−7^). Together, this comparison showed that the binding of C protein to SLBs was dependent on the presence of charged POPS (Figs 2C and S4). Interestingly, the ΔD upon C protein adsorption to the POPC/POPS SLBs was very low (3.8**×**10^−7^, s.d 1.4**×**10^−6^) indicating that the C protein forms a very rigid layer when adsorbed to the membrane [59]. This, in turn, indicates that the C protein does not bind as non-specific aggregates as these would have changed the viscoelastic properties of the system.

To probe the electrostatic nature of the initial C protein recruitment to the membrane, the C protein was injected in a buffer containing 1 M NaCl on SLBs preequilibrated with the same buffer. After reequilibrating the SLBs with TBS, the ΔF value was on average −0.6 Hz (s.d 0.3 Hz) compared to the pre 1 M NaCl equilibration showing that no C protein bound to the SLBs (Figs 2C and S4). The difference between injection on pure POPC SLBs and on POPC/POPS SLBs in 1M NaCl was not statistically significant (p-value 0.13). To test if the C protein membrane contacts are purely electrostatic, C protein was bound to SLBs in TBS, and then washed with 1 M NaCl buffer either right after equilibration or after a 2 h post-equilibration incubation. A wash with 1M NaCl resulted in what is referred to as “buffer shift” in the F and D values, which was reversed when moving back to the original buffer [60]. In both cases, only partial detachment of the C protein from the SLBs was detected with the final ΔF value being −11.9 Hz in the immediate wash experiment, and −11.3 in the 2h-incubation experiment (s.d. 1.9 and 1.5 Hz, respectively) (Fig 2D). The ΔF values were significantly different from zero with p-values of 0.013 (immediate wash experiment) and 0.009 (2h-incubation experiment). However, they were not significantly different between the experiments with a p-value of 0.10. This shows that initial membrane recruitment of C protein is strongly dependent on its interactions with the negatively-charged lipid head groups. However, once bound, the C protein-membrane interaction is complemented with non-electrostatic interactions such as membrane insertion or protein oligomerization within the bound layer. Since the amount of detached protein is not dependent on incubation time, it suggests that the protein´s non-electrostatic organization on the membrane occurs concurrently with adsorption.

When the same measurements were performed with the C_**18–93**_ construct, similar results to the wild-type C were obtained. The C_**18–93**_ protein injection caused a 24.1 Hz drop in ΔF (s.d 0.8 Hz) with the final ΔF value being significantly different from the C protein binding to pure POPC experiment (p-value 6.04**×10**^**−7**^) (**Figs 2C and S4**). When the bound C_**18–93**_ was washed with high-salt buffer, mass was retained on the SLB with a final ΔF value of **−**7.3 Hz (s.d. 0.2 Hz) (**Figs 2D and S4**). The final ΔF value was significantly different from zero (p-value 0.0002). Additionally, the final ΔF value was not significantly different from the full-length C protein experiments (p-values 0.079 and 0.066 for the comparison to the immediate wash and 2h incubation experiments respectively). This showed that the deletion of the N-terminus did not significantly alter membrane binding of the C-protein.

### C protein inserts into membranes

To investigate if the C protein membrane binding includes insertion into the membrane after the initial electrostatic binding, we used Langmuir-Blodgett trough monolayer experiments. In this approach, the insertion of a protein into a lipid monolayer can be detected by following the pressure (π) of the monolayer after protein injection into the aqueous subphase, with increases in π corresponding to protein injection [60,61]. We formed monolayers of pure POPC or POPC/POPS mixture (60:40%, w/w) and monitored the π value of the monolayers following a C or C_18–93_ injection into the subphase. The π increase (Δπ) between the starting (π_0_**)** and ending pressures were measured at different π_**0**_ values. The regression between Δπ and π_0_ was used to determine the maximum injection pressure (MIP) of the proteins.

**Fig 3:**
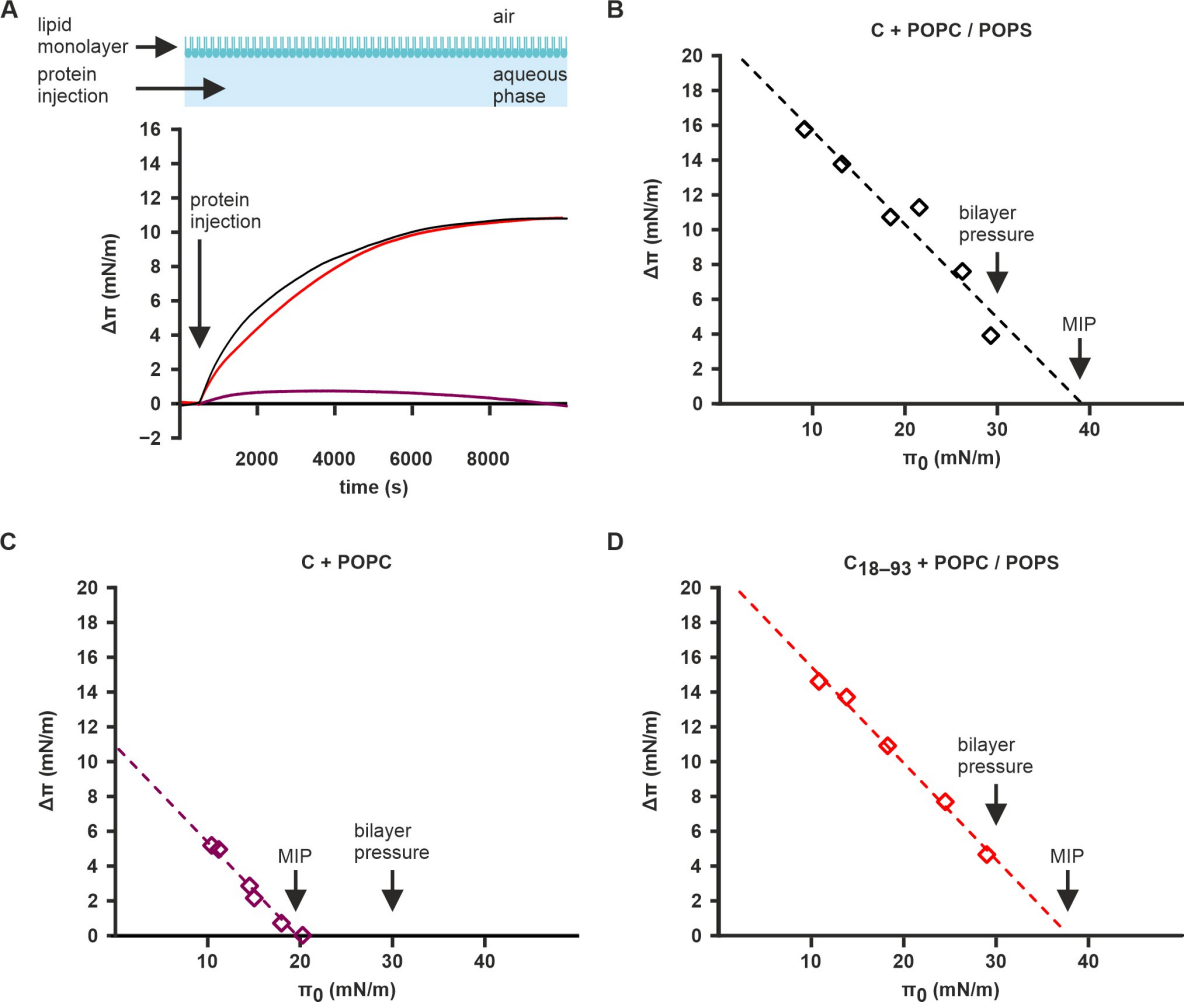
C protein inserts into membranes with negatively-charged lipids. **A** Representative Δπ curves for C protein injected with a POPC/POPS (black) or POPC (purple) monolayer, and C_18–93_ protein injected in with a POPC/POPS monolayer (red) at π_0_ values of 18.1±0.2 mN/m**. B**, **C, D** MIP curves of C protein with POPC/POPS (**B**) and pure POPC (**C**) monolayers, and C_18–93_ with POPC/POPS monolayers (**D**). The pressure of a bilayer (∼30 mN / m) is indicated along with the MIP [62–64]. Data information. In (**A**), π is zeroed to π_0_. The Individual measurements in (**B**), (**C**), and (**D**) are shown as diamonds. The R^2^ values in (**B**), (**C**), and (**D**) are 0.9344, 0.9818, and 0.9935, respectively.

Injection of full-length C into the subphase led to sharp π increases at all tested π_0_ values in monolayers containing 40% POPS (Figs 3A and S5). The MIP of full-length C was 39.1 mN/m, which is remarkably higher than the pressure of a lipid bilayer (~30 mN/m), suggesting that C is capable of inserting into bilayers such as the ER membrane (Fig 3B) [62–64]. Contrastingly, in the absence of POPS, smaller π increases were detected, and the Δπ value was zero at π_0_ of 20.20 mN/m (Figs 3A and S5). Furthermore, the MIP value of full-length C in pure POPC monolayers was 19.7 mN/m, which is notably smaller than the pressure of a lipid bilayer showing that negatively-charged lipids such as POPS are needed for C protein membrane insertion at physiological membrane pressures (Fig 3C) [62–64]. The C_**18–93**_ protein behaved similarly to the wild-type C in POPS-containing monolayers with a MIP of 37.7 mN/m (Figs 3A and S5). This corroborates the similar QCM-D results between C and C_**18–93**_, and shows that the N-terminus is not needed for C protein membrane insertion.

### Membrane-bound C protein can recruit TBEV genomic RNA

**Fig 4:**
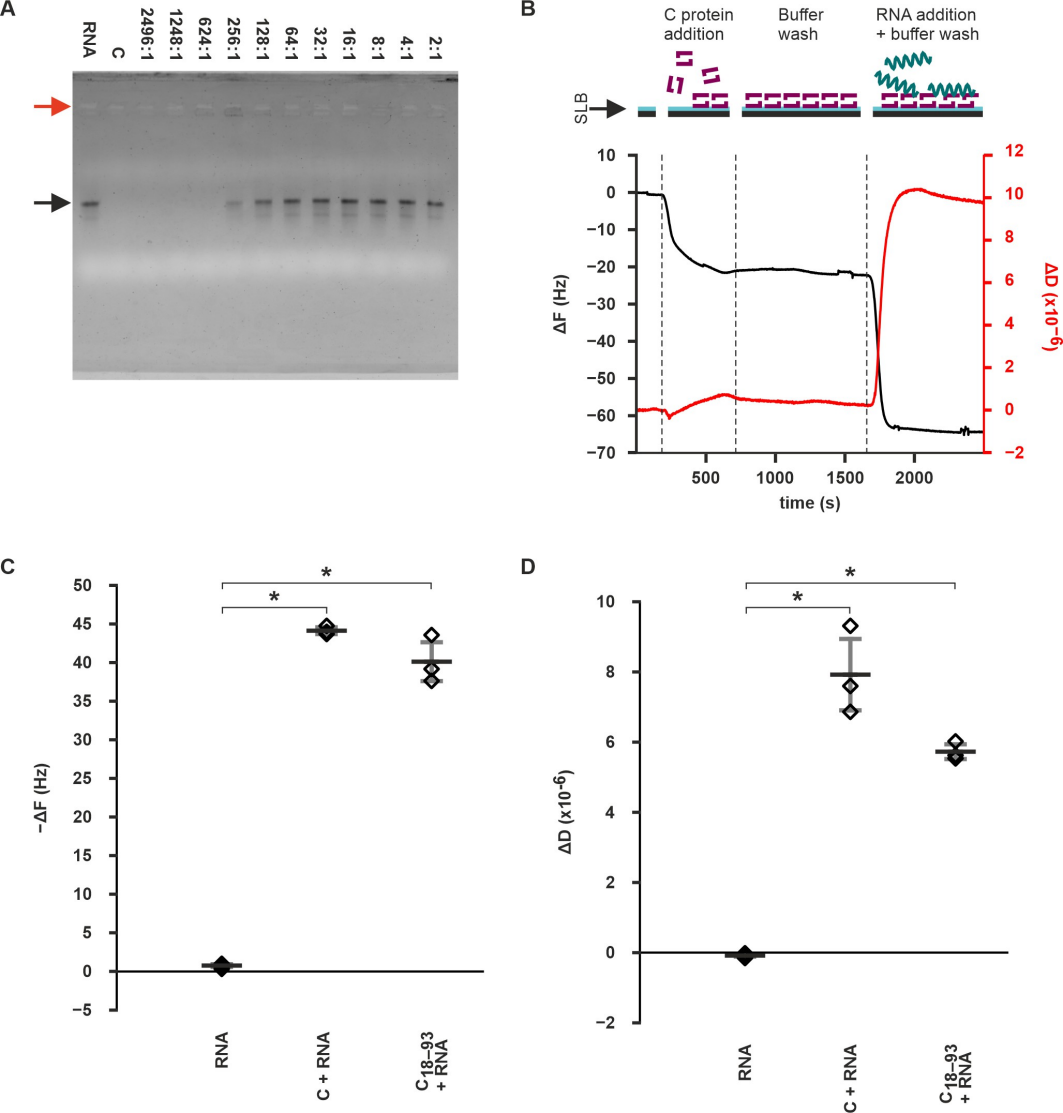
C protein can recruit RNA in solution and when bound on SLBs. **A** GEMSA gel showing C protein binding to RNA in solution. RNA refers to only RNA, and C to only C protein corresponding to the highest C protein concentration used. The C to RNA molar ratio is indicated. The black arrow indicates the bands of freely-migrating RNA, and the red arrow the wells. On lanes 624:1 and 256:1, RNA has been immobilized in the wells. **B** Representative QCM-D curves from an RNA-binding experiment on POPC/POPS SLBs with bound C protein. The ΔF and ΔD have been zeroed to equilibrium values after SLB formation. **C, D** Quantitation of ΔF (**C**) and ΔD (**D**) changes upon RNA insertion on SLBs pretreated with C, C_18–93_, or no protein. Data information. Data in C and D are shown as the mean of 3 repeats with s.d. represented by the error bars. Individual measurements are represented by the diamonds. Statistical significance (p < 0.05) is indicated with an asterisk. See the main text for the p-values.

Since RNA binding is crucial for the NC assembly, we investigated the C protein’s ability to perform this function. First, we confirmed the binding in solution using *in vitro* transcribed TBEV genomic RNA with a gel electrophoretic mobility shift assay (GEMSA). Using a C to RNA molar ratio of 624:1 fully prevented the RNA from migrating normally indicating binding (Fig 4A).

C protein presumably interacts simultaneously with membranes and RNA, and we therefore used QCM-D to study the C protein’s RNA recruitment capability whilst membrane-bound. In these experiments, C protein was essential for the recruitment of TBEV genomic RNA on SLBs (Fig 4B–4D). Injecting 5 μg of *in vitro* transcribed TBEV genomic RNA on SLBs which had bound C protein resulted in an average ΔF of −44.2 Hz at equilibrium (s.d. 0.5 Hz) showing RNA binding (Fig 4B and 4C). In contrast, the ΔF value with SLBs with no prebound C remained close to zero after RNA injection, with an average equilibrium ΔF of −0.6 Hz (s.d. 0.3 Hz) indicating no binding of mass on the SLBs (Figs 4C and S6). The equilibrium ΔF value difference between C protein-containing and protein-free SLBs was statistically significant (p-value 1.34×10^**−**6^) (Fig 4C). Interestingly, the ΔD value also increased to 7.92×10^**−**6^ on average after RNA binding (s.d. 1.03×10^**−**6^) in the protein-treated SLBs, indicating that the RNA did not bind as a rigid uniform layer but rather as a flexible assembly (Fig 4B and 4C). Without prebound C protein, the ΔD did not increase and remained at an average −8.8×10^−8^ at equilibrium (s.d. 5.4×10^−8^) (Figs 4C and S6). The equilibrium ΔD value difference between the experiments with and without prebound C protein was statistically significant (p-value 0.008) (Fig 4D). These data show that membrane-bound C protein is capable of recruiting TBEV genomic RNA at the membrane, suggesting that this also happens in the context of NC assembly.

When the SLB RNA-binding experiments were repeated with C_18–93_, the results were similar as with full-length C. After RNA injection on C_**18–93**_-treated SLBs, the ΔF value dropped to **−**40.1 Hz on average and the ΔD value increased to an average of 5.7×10^−6^ (s.d. 2.5 Hz and 2.1×10^−7^, respectively). The difference to the no protein experiment for both ΔF and ΔD was significant (p-values 0.002 and 0.0007, respectively) (Fig 4C and 4D). While the ΔF and ΔD values in the C_**18–93**_ experiments were closer to zero than in the wild-type C experiments, the differences were not statistically significant (p-values 0.156 and 0.097 respectively).

### TBEV virions contain negatively-charged phosphatidylserine

If the binding of C protein to negatively-charged lipid head groups is important for particle assembly, it is likely that these lipid species are incorporated into the virion during the budding into the ER. To investigate this possibility, we used UHPLC-QTOF-MS to characterize the TBEV lipid content of purified virions. The average integrated peak area of each investigated lipid species was compared between TBEV-containing samples, and buffer-only mock samples. Significantly higher integrated peak areas were interpreted as the presence of the species in the TBEV preparation, instead of noise caused e.g. by buffer or instrument contamination.

**Fig 5:**
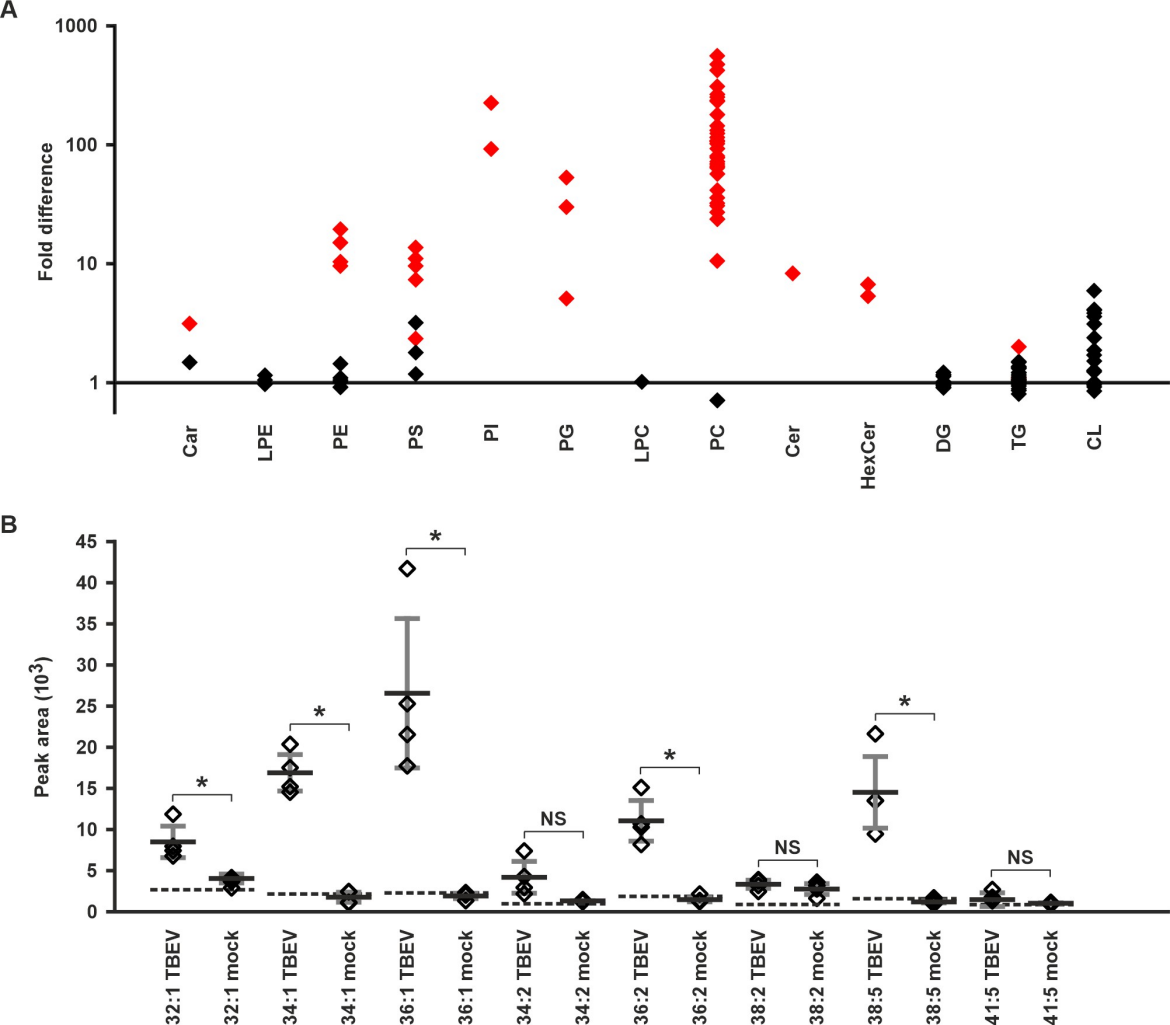
Mass spectrometric characterization shows that TBEV virions contain multiple lipid species. **A** Fold-increases of the integrated peak areas of the analysed lipid species in TBEV versus buffer samples. **B** Integrated peak areas of the detected PS species in TBEV virions and mock samples. The background peak level of each lipid species from a blank run is indicated with the dotted line. Data information: Data in A are shown as the ratio of the average integrated peak areas of TBEV virions divided by the buffer controls from four technical repeats. Data in B are shown as the averages of four technical replicates with the horizontal bar representing the mean and the error bars representing the s.d.. Individual species (**A**) or measurements (**B**) are represented by the diamonds. Significance in **A**, and **B** is indicated when p < 0.05 either in red (A) or with an asterisk (B). For the p-values, see S1 Table.

Multiple lipid species of different classes were detected in the TBEV samples (Fig 5A and S1 Table). The virions contained lipids from two negatively-charged classes, phosphatidylserine (PS) and phosphatidylglycerol (PG) (Fig 5A and S1 Table). In addition, neutral carnitine fatty acids (Car), phosphatidylethanolamines (PE), PIs, phosphatidylcholines (PC), ceramides (Cer), hexosylceramides (HexCer), and triglycerides (TG) were detected (Fig 5A and S1 Table). The analysed lipid classes from the in-house database that were not found in virions were lyso-PE (LPE), lyso-PC (LPC), diglyceride (DG), and cardiolipin (CL) (Fig 5B and S1 Table). The virions contained multiple PS species: 32:1, 34:1, 36:1, 36:2, and 38:5 (number of carbons:number of double bonds) (Fig 5B and S1 Table). This confirms the biological relevance of using PS lipids in the model membrane experiments, especially since a total carbon to double bond ratio of 34:1 corresponds to POPS. As C protein was shown not to bind to PE, PI, and PC in the liposome co-sedimentation assays (see above), these lipids are likely incorporated into the virion without direct interaction with the C protein. Both of the negatively-charged lipids, PS and PG may be incorporated into the virion via direct interactions with the C protein, as we have shown that these interactions are charge, but not head group dependent in the liposome co-sedimentation assay. PS and PG interactions may be important for the coordination of NC assembly.

## Discussion

Our unique combination of biophysical methods and model membrane systems allowed us to show that negatively-charged lipids are required for TBEV C protein membrane interactions, and that the protein inserts into membranes. The methods allowed us to demonstrate RNA recruitment onto membranes by the C protein, supporting that the NC assembly occurs on the ER membrane [7]. We show that residues 1–17 are not required for these interactions, and that membrane binding and RNA recruitment can occur independently of any other host or viral proteins. Additionally, we confirm the biological relevance of the interactions with negatively-charged lipids observed in the biophysical experiments by showing that negatively-charged PS and PG lipids are incorporated into the virion. PS has also been found previously in the lipid composition of West Nile virus [47]. Our findings on C protein membrane binding properties agree with previous observations from TBEV and other flaviviruses [40–42,65–67].

N-terminally truncated flaviviral C proteins have been shown to be assembly competent and *in vitro*, able to bind RNA, which is consistent with our results with N-terminally truncated TBEV C protein [41,68–70]. Instead, a role for the N-terminus may be in the reported modulation of host responses to infection [39,71–78]. The membrane insertion directly detected in our experiments is central to C protein function. Other studies have found that deletions in the hydrophobic region of the α_2_ helix significantly impair particle assembly [41,69,70]. In the light of this evidence, we consider that the α_2_ helix could be responsible for membrane insertion [40–42,65].

While it is clear that the C protein can perform both membrane binding and insertion as well as RNA binding simultaneously, the mechanism for this remains unclear. Based on the QCM-D results, the membrane binding followed two different kinetics, and after binding and insertion, only half of the C protein could be removed by a high NaCl wash (Figs 2B and 4B). This suggests the formation of higher-order oligomers on the membrane. Most probably one set of dimers are recruited for instance by the α_4_–α_4_ interface by charge interactions and insert into the membrane. Then they bind additional C protein molecules in another orientation, but they cannot insert due to steric hindrance which leaves their α_4_–α_4_ interface or other charged surfaces exposed for RNA recruitment. Only dimers have been detected in TBEV virions by cross-linked mass spectrometry, but in order to form the NC, it is likely that higher-order C protein interactions are needed [37]. In cross-linking experiments with purified TBEV C protein, a population of trimers were detected which shows that higher-order oligomerisation is possible [34]. However, we cannot rule out that our C protein preparation has microheterogeneity despite its appearance in SEC, which could cause the partial insertion observed. Further functional studies are needed to determine the orientation, conformation and multimerisation status of C on the membrane.

Another factor that could influence the C protein interaction with negatively-charged lipids, would be its phosphorylation state. In West Nile virus, C protein, phosphorylated by protein kinase C, is imported into the nucleus through binding to importin α. The nuclear import leads to localization in the nucleolus where C binds to HDM2 starting apoptosis [79]. Phosphorylation decreases the cytoplasmic C protein amount, and reduces binding to RNA [80], thus decreasing the pool of protein available for NC assembly. TBEV C protein is also transported to the nucleolus, likely through phosphorylation-mediated binding to importin α as well [34,53]. Likely residues for phosphorylation are S19, S65 S83. Since S83 is present on the **α**_**4**_ helix, phosphorylating it could repel the negatively-charged membrane and prevent binding, whereas dephosphorylation would promote assembly, increasing the propensity of C to bind to both membranes and RNA in TBEV. Such a mechanism for controlling the binding to negatively-charged lipids has recently been reported for herpesvirus nuclear egress complexes [81].

Recently, it has been shown that the neutralization of the C protein surface positive charge is important for RNA binding in the distantly-related Dengue virus (DENV) [82]. The recruitment of C protein to the membrane, that we demonstrate is dependent on negatively-charged head groups, provides a biologically relevant mechanism for charge neutralization on the C protein surface that interacts with the lipids. The remaining surface charge can be then neutralized by RNA recruitment. Therefore, negatively-charged ER lipid patches may act as the nucleation sites for NC assembly by binding the C protein that subsequently recruits the genomic RNA. PS, which we detected in the virion envelope, is also a major component of the ER [83]. ER lipid microdomains are most prevalent at the contact sites between the ER, lipid droplets, and mitochondria (rich in PG) [83,84]. These sites are associated with extensive exchange of lipids between organelles [83,84]. Mitochondria and lipid droplets are often observed in close proximity to the ER replication sites of TBEV, DENV and Zika virus [49,50,85]. Additionally, the recruitment of the C protein to lipid droplets has been suggested to be important for DENV assembly [51,86]. As flavivirus infections cause changes in the host cell lipid metabolism, it is possible that this leads to an increase in PS and PG content in the ER. The observed frequent association of mitochondria and lipid droplets with flavivirus replication sites as well as the presence of PS and PG in the TBEV envelope maybe the results of this [46,47,86].

In conclusion, we have determined that NC assembly is most likely membrane-associated, involving specific lipids. Although often ignored in virus structure and assembly, the importance of specific lipid species is gradually being recognised in flavivirus assembly, including the recent discoveries of ordered lipids in flavivirus membranes coordinated by the E and M membrane helices [6,87–90]. Our results provide a starting point for further structural and mechanistic studies on the role of lipid interactions in TBEV NC assembly.

## Materials and methods

### Homology modelling

A homology model of the full-length TBEV C protein from the Kuutsalo-14 strain (GenBank: MG589938.1) was generated using the I-TASSER server (https://zhanggroup.org/I-TASSER/) with the truncated TBEV C protein structure (PDB: 7YWQ) as a template [34,54,55,57]. The electrostatic surface potential of the homology model was calculated using the APBS-PDB2PQR software suite (https://server.poissonboltzmann.org/) using the CHARMM force field and visualized with ChimeraX [91–94].

### Cloning and protein purification

Full-length TBEV C protein (residues 1–96) and a truncated C protein (C_**18–93**_, containing residues 18–93) from the Kuutsalo-14 strain (GenBank: MG589938.1) with an N-terminal 6x His + Small Ubiquitin-like Modifier (SUMO) tag were cloned into pET28 plasmid as a commercial service by DNA Dream Lab (Helsinki, Finland) [57]. The proteins were expressed in *E*. *coli* ArcticExpress cells. The cells were grown o/n at 37°C with 225 RPM shaking in lysogeny broth (LB, 1% w/v tryptone, 0.5% w/v yeast extract, 85 mM NaCl) containing 50 μg/ml kanamycin and 10 μg/ml gentamycin. Cells were inoculated into fresh LB without antibiotics, and grown at 30°C with 225 RPM shaking until OD600 reached 0.5. The cultures were moved to +12°C with 225 RPM shaking for an hour and induced with isopropyl β-d-1-thiogalactopyranoside (IPTG, final concentration 1 mM). The induced cultures were grown for 24 h at 12°C with 225 RPM shaking and harvested by centrifuging at 3000 × *g* for 20 min at +4°C. The supernatant was discarded, and the pellets stored at −80°C until needed.

The frozen pellets were resuspended on ice in 300 mM NaCl, 50 mM Tris, 375 mM L-arginine, 20 mM imidazole, pH 7.5 containing Pierce protease inhibitors (Thermo Fisher, A32965). The cells were lysed with an Emulsiflex apparatus (Avestin Inc., Ontario, Canada) operated at 15 000 psi for 10 min. The cell lysates were precleared for 30 min at 20 200 × *g* at + 4°C. The resulting supernatant was further cleared by centrifuging at 96 600 ×*g* for 1.5 h at +4°C. The proteins were captured with nickel immobilized metal affinity chromatography using a 5 ml HisTrap FF column (Cytiva, Massachusets, USA) and a linear imidazole gradient from 0 mM to 1000 mM over 25 column volumes. The purified proteins were buffer-exchanged into 300 mM NaCl, 50 mM Tris, pH 7.5 with PD 10 columns (Merck, New Jersey, USA) using the manufacturer’s protocol. The SUMO tags were cleaved using in-house-produced SUMO protease o/n (full-length C) or for 48 h (C_**18–93**_) at room temperature (RT) with gentle agitation. The cleaved tags were removed by cation exchange chromatography using a 1 ml HiTrap SP HP column (Cytiva) with a linear NaCl gradient from 300 mM to 1000 mM over 25 column volumes. SEC was used to buffer exchange the samples into 300 mM NaCl, 50 mM Tris, pH 7.5 using a Superdex 200 10/300 GL column (Cytiva). The peak fractions were collected, aliquoted, and stored in −80°C. The aliquots were characterized with SDS-PAGE and immunoblotting (see below), the protein concentrations were measured with a QFX fluorometer (DeNovix, Delaware, USA) using a Qubit Flex kit (Thermo Fisher, Massachusetts, USA), and the A_260_/A_280_ ratios of the samples were measured using a Nanodrop instrument (Thermo Fisher).

### SDS-PAGE and immunoblotting

Samples were mixed with Laemmli sample buffer, incubated for 5 min at 95°C, and proteins were resolved using electrophoresis in 4–20% polyacrylamide gels (Bio-Rad, California, USA). Proteins were visualized using Coomassie blue staining or by immunoblotting. For immunoblotting, the proteins were transferred onto nitrocellulose membranes (GE Healthcare, Illinois, USA) using a Trans Blot turbo apparatus (BioRad) using the manufacturer’s protocol. The membranes were blocked using 5% (w/v) milk in 150 mM NaCl, 20 mM Tris, 0.1% TWEEN 20, pH 7.6 (TBST) for 30 min at RT with gentle rocking and washed with TBST. Membranes were incubated with the primary anti-C antibody (1:1000 dilution in TBST containing 5% milk) [52]. After incubation, the membranes were washed 3 times with TBST for five minutes with gentle rocking. The secondary antibody was diluted 1:10 000 in TBST, added on the membranes, and the membranes were incubated for 30 minutes at RT with gentle rocking. The secondary antibody used was IRDye 680 RD goat anti-rabbit IgG (Li-COR). After incubation, the membranes were washed with TBST and visualised using an Odyssey imager (Li-COR).

### Lipids

The following lipids were purchased from Avanti Polar Lipids (Alabama, USA) as lyophilized powder: DOPC (850375), DOPE (850725), porcine BTLE (131101), POPS (sodium salt, 840034), SM (N-[dodecanoyl]-sphing-4-enine-1-phosphocholine, LM2312), ovine Chol (700000), bovine liver L-α-PI (840042), porcine brain PI(4,5)P2 (ammonium salt, 840046), and POPC (850457). Bovine brain GalCer (C4905) were purchased from Sigma-Aldrich (Missouri, USA) as lyophilized powder. Lissamine rhodamine B 1,2-dihexadecanoyl-sn-glycero-3-phosphoethanolamine (r-DHPE, triethylammonium salt, L1392) was purchased as a lyophilized powder from Thermo Fisher.

### Liposome co-sedimentation assay

The protocol was adapted from reference [95]. Liposomes of various lipid species (see Table 1) were prepared by diluting total of 0.5 mg of lipids in a 10:3 mixture of chloroform and methanol in glass tubes. Lipid films were prepared by drying the lipids under a gentle nitrogen stream while rotating the glass tubes. The lipids were further dried under a gentle nitrogen stream for 30 min. The lipids were rehydrated to 1 mg/ml by adding 500 μl 150 mM NaCl, 50 mM Tris, pH 7.6 (TBS) and by incubating at RT for 30 min. The liposomes were sonicated with a bath sonicator for 20 s (Transsonic T310, Elma Schmidbauer, Singen, Germany) and either used immediately, or stored at +4°C for up to two days.

Fresh aliquots of full-length C protein were thawed on ice and centrifuged for 10 min at 20 000 × *g* at + 4°C, and the supernatant collected. The protein was incubated either with experimental liposomes or TBS for 5 min at RT with a final protein concentration of 7 μM and a final lipid concentration of 500 μg/ml. The liposomes were pelleted by centrifuging for 20 min at 100 000 × *g* at RT. The supernatants were collected, and the pellets resuspended in 50 μl TBS. Both the supernatants and the pellets were analysed with SDS-PAGE as above, and the fraction of C protein co-sedimenting with the liposomes was quantified using Fiji [96].

**Table 1:** Liposome compositions used for co-sedimentation assays.

Lipid species	composition (%, w/w)
DOPC/DOPE	60:40
DOPC/DOPE	50:50
BTLE	100
DOPC/DOPE/POPS	45:45:10
DOPC/DOPE/POPS	40:40:20
DOPC/DOPE/POPS	35:35:30
DOPC/DOPE/POPS	30:30:40
DOPC/DOPE/Chol	40:40:20
DOPC/DOPE/SM	40:40:20
DOPC/DOPE/PI	40:40:20
DOPC/DOPE/PI(4,5)P_2_	55:40:5
DOPC/DOPE/GalCer	40:40:20

### Liposome flotation assay

The protocol was adapted from reference [97]. Liposomes containing either DOPC/DOPE/r-DHPE/POPS (15:14:1:40%, w/w) or DOPC/DOPE/r-DHPE (60:39:1%, w/w) were prepared as for the co-sedimentation assay, except that they were rehydrated in TBS containing 0.3 M sucrose. Purified full-length C protein was cleared as above and incubated with TBS containing 0.3 M sucrose or the liposomes (protein concentration 7 uM, lipid concentration 415 mg/ml) for 10 min at RT in 1 ml ultracentrifuge tubes. The step gradient was created by adding 60% sucrose in TBS (w/w) to yield a final sucrose concentration of 40% (w/w) to the protein-liposome mixture at the bottom of the tube. This was overlaid first with 25% (w/w) sucrose in TBS, followed by a layer of TBS. The samples were centrifuged for 30 min at 259 000 × *g* at + 4°C. Samples (100 μl aliquots) were collected from the TBS phase (fraction 1), the liposome-containing TBS-25% sucrose phase boundary (fraction 2), the 25% (w/w) sucrose-containing phase (fraction 3), and the 40% (w/w) sucrose-containing phase (fraction 4). SDS was added to the samples to yield a final concentration of 0.1% (w/v), and the samples were mixed at RT. The relative protein content of the fractions was quantitated with SDS-PAGE and Fiji (see above). The relative lipid content of the fractions was quantitated by diluting the samples 1:50 in TBS and measuring the r-DHPE fluorescence using a VICTOR Nivo plate reader (PerkinElmer, Massachusetts, USA) with excitation set at 570 nm and emission set at 595 nm.

### RNA synthesis and electrophoretic mobility shift assay

Full-length TBEV genomic RNA was produced from an infectious clone based on the TBEV 93/783 sequence (GenBank: MT581212) using *in vitro* transcription. Linearized DNA containing the clone was transcribed using the mMESSAGE mMACHINE SP6 Transcription kit (Thermo Fisher) according to the manufacturer’s protocol. RNA was purified by LiCl precipitation according to the transcription kit protocol. RNA concentration was measured using a nanophotometer (DeNovix).

Transcribed RNA (300 ng) was mixed with C protein at molar ratios ranging from 1:2 to 1:2496 in 150 mM NaCl, 50 mM Tris, pH 7.5 prepared with RNAse-free reagents (RNA buffer) and incubated on ice for 20 min. The samples were mixed with loading dye (final composition: 60% v/v formamide, 0.0133% w/v bromophenol blue, 0.0053% v/v xylene cyanol, 0.72% w/v Tris, 0.37% w/v boric acid, 6.6 mM EDTA) and analysed with electrophoresis on a 0.5% agarose gel containing SYBR Green I (Thermo Fisher) for 50 min at 70 V.

### SLBs and QCM-D

The protocol was adapted from reference [60]. Liposomes containing either pure POPC, or a mixture of POPC and POPS (60:40% w/w) were prepared as above in 20 mM citric acid, 50 mM KCl, 0.1 mM EDTA, pH 4.5 (CKB). The liposomes were homogenized by extruding 11 times through a 50 nm polycarbonate filter (Nuclepore Track-Etched Membranes, Whatman, Maidstone, UK) using a Mini Extruder instrument (Avanti Polar Lipids). QCM-D experiments were conducted with an X4 instrument equipped with a flow chamber (AWSensors, Valencia, Spain) using wrapped 14 mm 5 MHz, Cr/Au-SiO_2_ polished QCM-D sensors (AWSensors) at +23°C. The sensors were incubated overnight in 2% SDS and cleaned with a UV-ozone cleaner (Bioforce Nanosciences, Iowa, USA) for 30 min before use. Frequency and dissipation were monitored for overtone 3, although they were measured for overtones 1, 5, 7, 9, and 11 as well. The chambers were filled with CKB, and the SLBs were formed by injecting 150 μl of the liposomes at a concentration of 0.1 mg/ml. After the formation of the SLBs, TBS was injected until the system reached equilibrium. For the 100% POPC SLBs, 7.7 μg of C protein in 100 μl TBS (corresponding to a concentration of 7 μM) was injected. For the 60% POPC/40% POPS SLBs, full-length C or C_**18–93**_ was injected either as above or the SLBs were washed with 1000 mM NaCl, 50 mM Tris, pH 7.6 (HSQB) and C protein injected as above but in HSQB. After C protein injection, the chambers were washed with TBS until equilibrium. In the experiments where C protein was injected in TBS, the protein-containing SLBs were washed with HSQB either immediately after reaching equilibrium, or after 2h incubation. Similarly, in C_**18–93**_ experiments, the protein-containing SLBs were washed with HSQB immediately after equilibrium was reached. After HSQ wash, the SLBs were washed with TBS until equilibrium. For the RNA experiments, the SLBs were washed with RNA buffer either without protein addition, or after an injection of C or C_**18–93**_ as above. 5 μg of *in vitro* transcribed TBEV genomic RNA in 100 μl RNA buffer was injected, followed with a wash with RNA buffer, and a wash with TBS until equilibrium. The equilibrium ΔF and ΔD values for each step were calculated as the average of a 30 s period starting 400 s after the start of the final TBS wash.

### Lipid monolayer experiments

The protocol was adapted from reference [60]. Lipid formulations of either pure POPC or a 60:40% (w/w) mixture of POPC and POPS were prepared in chloroform (final lipid concentration 0.5 mg / ml). A Microtrough G1 system (Ø 53 mm x 4 mm, Kibron, Helsinki, Finland) was used for the experiments. The trough was covered to prevent evaporation, and the experiments were conducted at +21°C. The subphase was formed of TBS and constantly stirred with a magnetic stirrer during the measurement. π was measured with a Wilhelmy paper plate presoaked in TBS and calibrated for each measurement. The monolayers were formed by adding the lipid solutions on to the subphase in a dropwise manner and letting the chloroform evaporate. Once the initial π_0_ value of the experiment was reached, the monolayer was left to equilibrate for 30 min. Then, C protein or C_**18–93**_ in TBS was injected with a syringe under the monolayer via an injection port to reach the final protein concentration of 30 nM in the subphase. The π value was monitored until it plateaued. The π_0_ values were calculated as an average of the 30 s preceding the injection, and the final π values were calculated as the average of the 30 s surrounding the highest value reached. The MIP-values were determined by plotting Δπ against π_0_ and extrapolating the Δπ/π_0_ plot to the x axis.

### Cell culture

Human neuroblastoma SK-N-SH cells (gift from Prof. Olli Vapalahti, University of Helsinki) were maintained in low-glucose Dulbecco′s Modified Eagle′s Medium (DMEM, Sigma) supplemented with 10% foetal bovine serum (FBS, Gibco), 0.5 mg/ml penicillin, 500 U/ml streptomycin (Lonza Bioscience, California, USA), and 2 mM glutamine (Gibco, Waltham, MA, USA). The cells were maintained at +37°C in a 5% CO_2_ atmosphere.

### Virus propagation and titration

TBEV-Eu strain Kuutsalo-14 passage 1 (gift from Prof. Vapalahti) was used to infect confluent SK-N-SH cells using a multiplicity of infection of 0.003 [57]. The cells were washed twice with phosphate-buffered saline (PBS), virus was added in infection medium (low-glucose DMEM, 0.5 mg/ml penicillin, 500 U/ml streptomycin, 2 mM glutamine, 2% FBS, 35 nM rapamycin [98]), and incubated at +37°C in 5% CO_2_. At 72 h post-infection, the supernatant was collected, and centrifuged for 10 min at +4°C at 3800 × g. After centrifugation, the supernatant was collected and immediately purified (see below), or titered and stored at -80°C.

For titration, virus samples were serially diluted 10 fold in infection medium. Confluent SK-N-SH cells in 6-well plates were washed twice with PBS, and 200 μl of the virus dilutions or infection medium was added on the cells. The cells were incubated at +37°C for 1 h with gentle shaking every 5 min. After the incubation, 3 ml of minimum essential medium (MEM, Gibco) containing 0.5 mg/ml penicillin, 500 U/ml streptomycin, 2 mM glutamine, 2% FBS, and 1.2% Avicell was added to each well. The cells were incubated at +37°C in a 5% CO_2_ atmosphere for 4 days, after which the medium was removed, the cells washed with PBS, and fixed with 10% formaldehyde for 30 min at RT. The plaques were visualised by incubating the fixed cells with 0.5% crystal violet for 10 min and washing with water. The viral titre was expressed as plaque-forming units per ml (pfu/ml).

### Virus purification

The virus was precipitated from the cell supernatant by adding 8% (w/v) polyethylene glycol (PEG) 8000 (Sigma-Aldrich), incubating at +4°C with gentle mixing for 3 h, and pelleted at +4°C, at 10,500× *g* for 50 min. The supernatant was discarded, and the pellets were carefully washed with 20 mM HEPES, 150 mM NaCl, 1 mM EDTA, pH 8.5 (HNE) and resuspended by incubating in HNE overnight at +4°C on an orbital shaker.

The dissolved pellets were loaded onto linear 10–70% sucrose gradients prepared in HNE. The samples were centrifuged at +4°C at 44,500 × *g* for 17 h and the virus-containing light-scattering bands were collected. The samples were buffer exchanged to HNE using Slide-A-Lyzer dialysis cassettes (MWCO 10 kDa; ThermoFisher) at +4°C for 1h with gentle agitation and the HNE was replaced with fresh HNE, and the dialysis continued overnight. The titre was determined using plaque titration.

### Mass spectrometry analysis of TBEV lipids

Purified virus (7×10^8^ pfu) in HNE or just HNE was mixed with methanol and chloroform (1:3:1 ratio of sample:methanol:chloroform), and vortexed for 30 s. Insoluble components were precipitated by centrifuging for 10 min at +4°C at 20 000 × *g* and the supernatants were stored in glass vials with a nitrogen atmosphere in -80°C.

Lipidomic profiling by UHPLC-QTOF-MS was performed at the Swedish Metabolomics Center in Umeå, Sweden. Prior to analysis the samples were transferred to microvials, evaporated under a stream of nitrogen and redissolved in 60 μl (2:1 v/v chloroform:methanol) including internal standards (tripalmitin-1,1,1-13C3, 16:0-d31 ceramide, 1,2-distearoyl-d70-sn-glycero-3-phosphocholine and 1,3-dioctadecanoyl-2-hydroxy-sn-glycerol-d5). The LC-MS analysis of the lipid extracts were performed on an Agilent 1290 Infinity UHPLC-system coupled to an Agilent 6546 Q-TOF mass spectrometer (Agilent Technologies, Waldbronn, Germany) as previously described reference [99].

The data were processed using Batch Targeted Feature Extraction algorithm within MassHunter™ ProFinder version B.10.02 (Agilent Technologies Inc., Santa Clara, CA, USA). An in-house database with exact mass and experimental retention times of lipids was used for identification.

### Statistical procedures

Statistical significance of the results were assessed either using one sample T-test (assessment of remaining F signal in 1M NaCl wash QCM-D experiments) or Welch’s T-test (all other experiments) performed in R 4.2.1.

## Supporting information

S1 Fig Structure and homology model of the TBEV C protein. **A, B** Ribbon representation of the lowest energy conformation of the truncated C protein (PDB: 7YWQ) (**A**) and the full-length C protein homology model (**B**) dimers from two angles [34]. The chains are coloured turquoise and purple, and the α_1_–α_4_ helices and the termini are labelled for one monomer. The residues truncated from the C_**18–93**_ construct are highlighted in magenta in (**B**). **C** Surface representation of the full-length C homology model dimer from the same angles as in **A** and **B**. The surfaces are coloured according to electrostatic potential according to the key (kT/e at 298 K, pH 7.0).
(TIF)

S2 Fig Biochemical characterisation of purified C protein. **A** SDS-PAGE analysis of the purified C protein. **B** Anti-C immunoblot analysis of the purified C protein. **C** SDS-PAGE analysis of the purified C_**18–93**_ protein. **A-C** M lanes show the molecular size marker and S lanes the protein preparations. The sizes of the molecular size marker bands and the C, and C_**18–93**_ protein band positions are indicated.
(TIF)

S3 Fig Liposome flotation assay. **A** Representative Coomassie-blue stained SDS-PAGE of fractions 1–4 from liposome flotation assays with no liposomes, liposomes containing 0% POPS and liposomes containing 40% POPS. The black arrow indicates the full-length C protein band and the red arrow indicates the lipids. **B**, **C**, **D** Densitometric quantitation of the C protein signal in the flotation fractions after SDS-PAGE. In the absence of liposomes (**B**), with liposomes containing 0% POPS (**C**), and liposomes containing 40% POPS (**D**). **E**, **F** Quantitation of the fraction of the total 595 nm r-DHPE fluorescence in the flotation fractions from experiments with liposomes containing 0% POPS (**E**), or 40% POPS (**F**). Data information: In **B**–**F**, data are shown as the averages of three replicates with the error bars representing the s.d.. Individual measurements are shown as diamonds. Data are normalized against the sum of the densitometric signal (**B**, **C**, **D**) or the sum of the fluorescence (**E**, **F**).
(TIF)

S4 Fig Representative QCM-D curves of the C and C_18–93_ proteins binding to SLBs. **A** Representative QCM-D curves from a C protein binding experiment on POPC SLBs. **B** Representative QCM-D curves from a C protein binding in 1M NaCl experiment on POPC/POPS SLBs. **C** Representative QCM-D curves from a C protein binding with a 1M NaCl wash after 2h incubation experiment on POPC/POPS SLBs. **D** Representative QCM-D curves from a C_18–93_ protein binding experiment on POPC/POPS SLBs. Data information: In each panel, the ΔF and ΔD have been zeroed to equilibrium values after SLB formation.
(TIF)

S5 Fig Δπ curves for C and C_18–93_ proteins injected at different Δ_0_ values shown for the same timescale as in Fig 3. A Δπ curves for C protein injected with POPC monolayers. B Δπ curves for C protein injected with POPC/POPS monolayers. C Δπ curves for C_18–93_ protein injected with POPC/POPS monolayers. Data information: The π is zeroed to the preinjection pressure. The π_0_ values for each curve are indicated.
(TIF)

S6 Fig Representative QCM-D curves of RNA binding to SLBs. **A** Representative QCM-D curves from an RNA binding without protein pretreatment experiment on POPC/POPS SLBs. **B** Representative QCM-D curves from an RNA binding with pretreatment with C_18–93_ experiment on POPC/POPS SLBs. Data information: In each panel, the ΔF and ΔD have been zeroed to equilibrium values after SLB formation.
(TIF)

S1 Table Integrated peak areas of the lipids analysed via UHPLC-QTOF-MS. (XLSX)
