# Supplementary figures and images for: Simultaneous membrane and RNA binding by tick-borne encephalitis virus capsid protein
Source: PLoS Pathog. 2023 Feb 14;19(2):e1011125. doi: 10.1371/journal.ppat.1011125 (PMC9970071; doi:10.1371/journal.ppat.1011125)

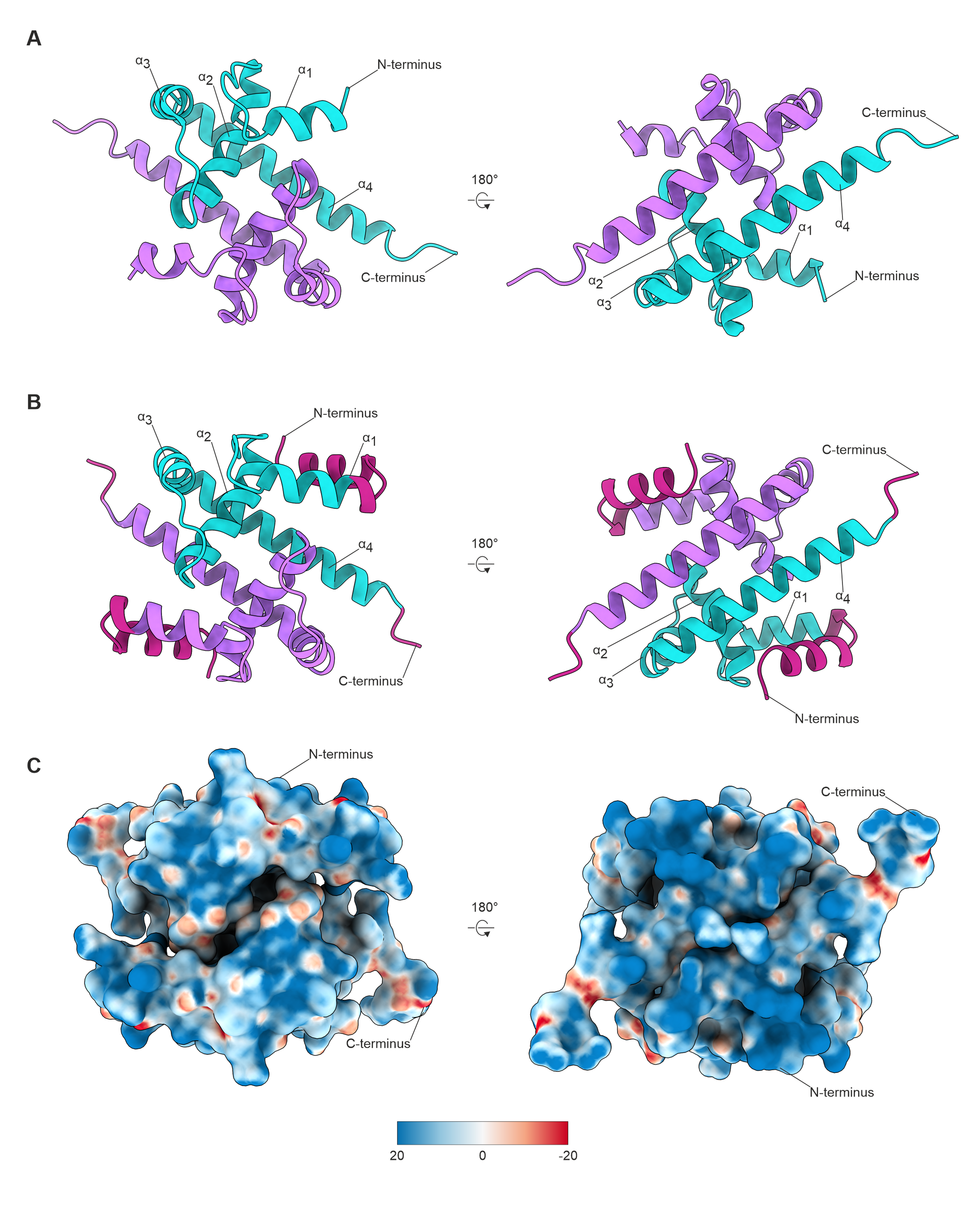

Supplement: S1 Fig — A, B Ribbon representation of the lowest energy conformation of the truncated C protein (PDB: 7YWQ) (A) and the full-length C protein homology model (B) dimers from two angles [34]. The chains are coloured turquoise and purple, and the α1–α4 helices and the termini are labelled for one monomer. The residues truncated from the C18–93 construct are highlighted in magenta in (B). C Surface representation of the full-length C homology model dimer from the same angles as in A and B. The surfaces are coloured according to electrostatic potential according to the key (kT/e at 298 K, pH 7.0). (TIF) [file ppat.1011125.s001.tif]

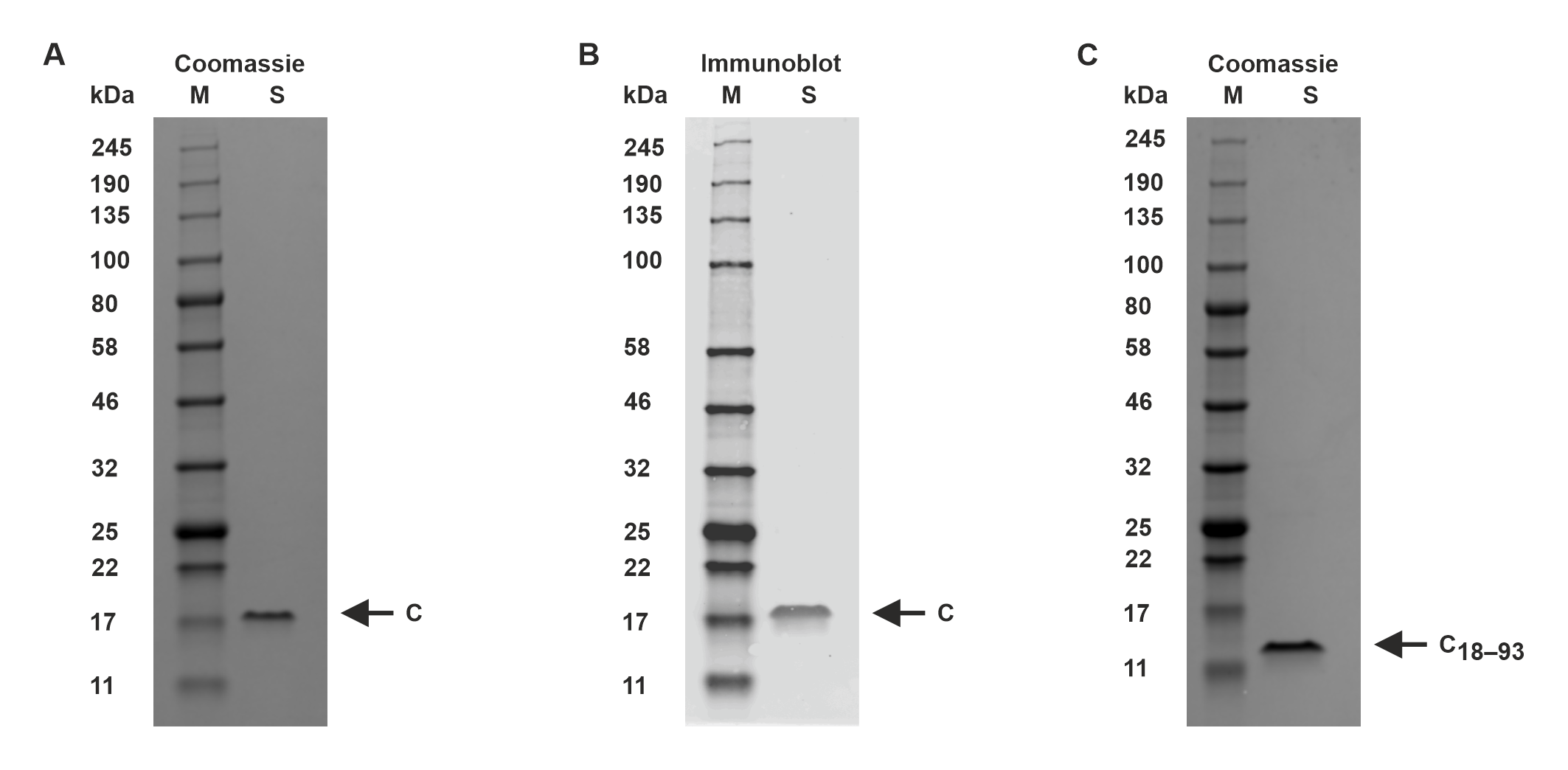

Supplement: S2 Fig — A SDS-PAGE analysis of the purified C protein. B Anti-C immunoblot analysis of the purified C protein. C SDS-PAGE analysis of the purified C18–93 protein. A-C M lanes show the molecular size marker and S lanes the protein preparations. The sizes of the molecular size marker bands and the C, and C18–93 protein band positions are indicated. (TIF) [file ppat.1011125.s002.tif]

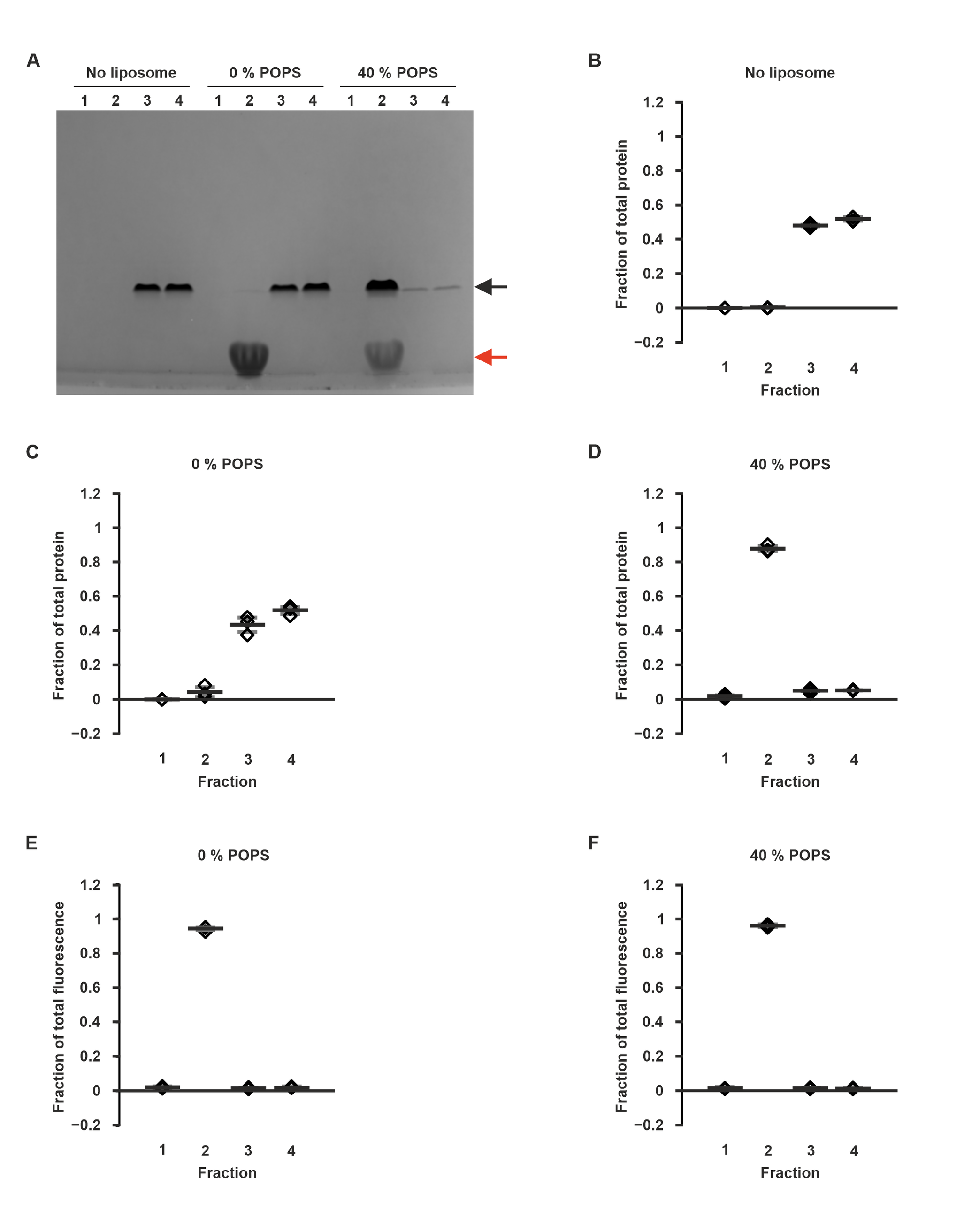

Supplement: S3 Fig — A Representative Coomassie-blue stained SDS-PAGE of fractions 1–4 from liposome flotation assays with no liposomes, liposomes containing 0% POPS and liposomes containing 40% POPS. The black arrow indicates the full-length C protein band and the red arrow indicates the lipids. B, C, D Densitometric quantitation of the C protein signal in the flotation fractions after SDS-PAGE. In the absence of liposomes (B), with liposomes containing 0% POPS (C), and liposomes containing 40% POPS (D). E, F Quantitation of the fraction of the total 595 nm r-DHPE fluorescence in the flotation fractions from experiments with liposomes containing 0% POPS (E), or 40% POPS (F). Data information: In B–F, data are shown as the averages of three replicates with the error bars representing the s.d.. Individual measurements are shown as diamonds. Data are normalized against the sum of the densitometric signal (B, C, D) or the sum of the fluorescence (E, F). (TIF) [file ppat.1011125.s003.tif]

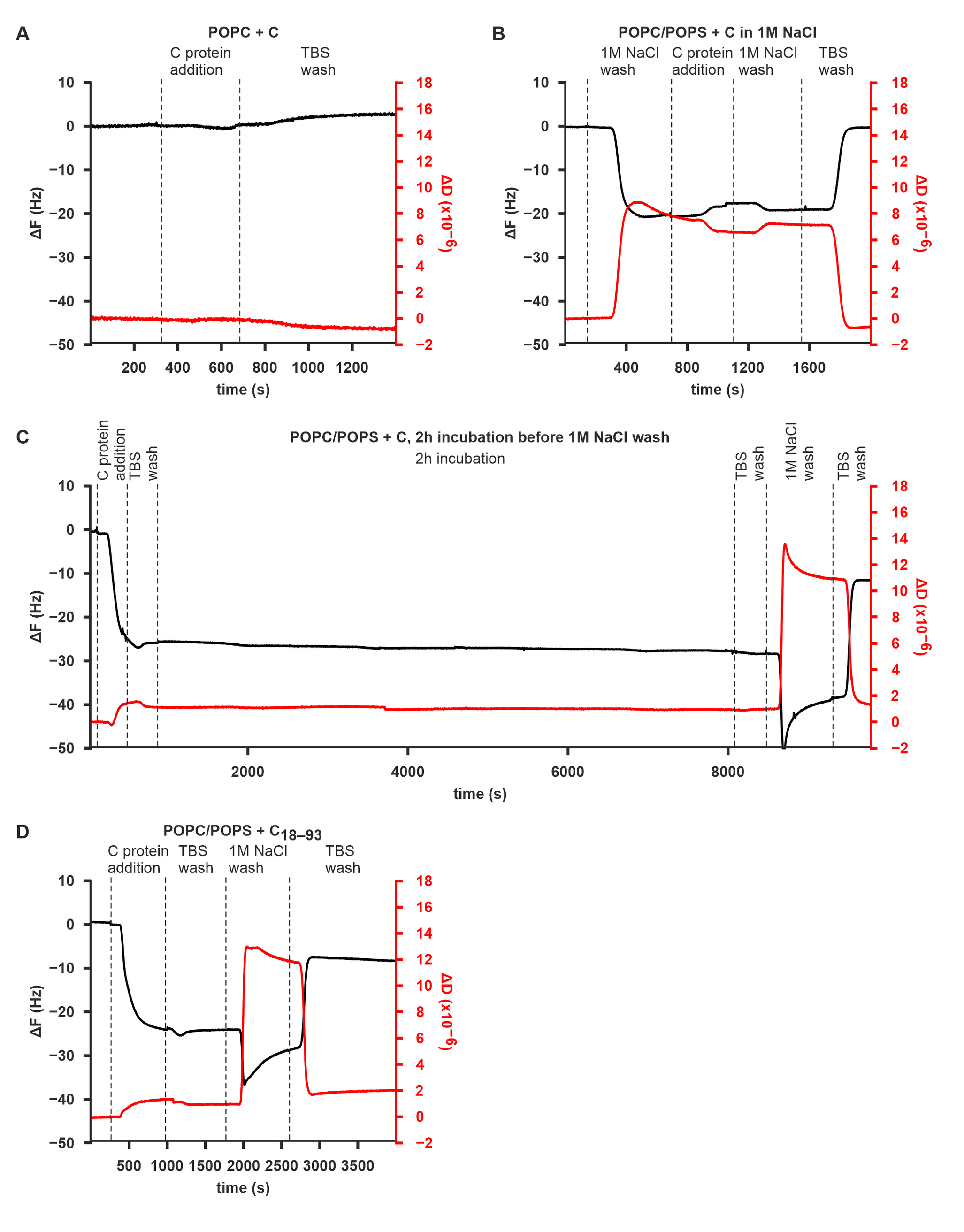

Supplement: S4 Fig — A Representative QCM-D curves from a C protein binding experiment on POPC SLBs. B Representative QCM-D curves from a C protein binding in 1M NaCl experiment on POPC/POPS SLBs. C Representative QCM-D curves from a C protein binding with a 1M NaCl wash after 2h incubation experiment on POPC/POPS SLBs. D Representative QCM-D curves from a C18–93 protein binding experiment on POPC/POPS SLBs. Data information: In each panel, the ΔF and ΔD have been zeroed to equilibrium values after SLB formation. (TIF) [file ppat.1011125.s004.tif]

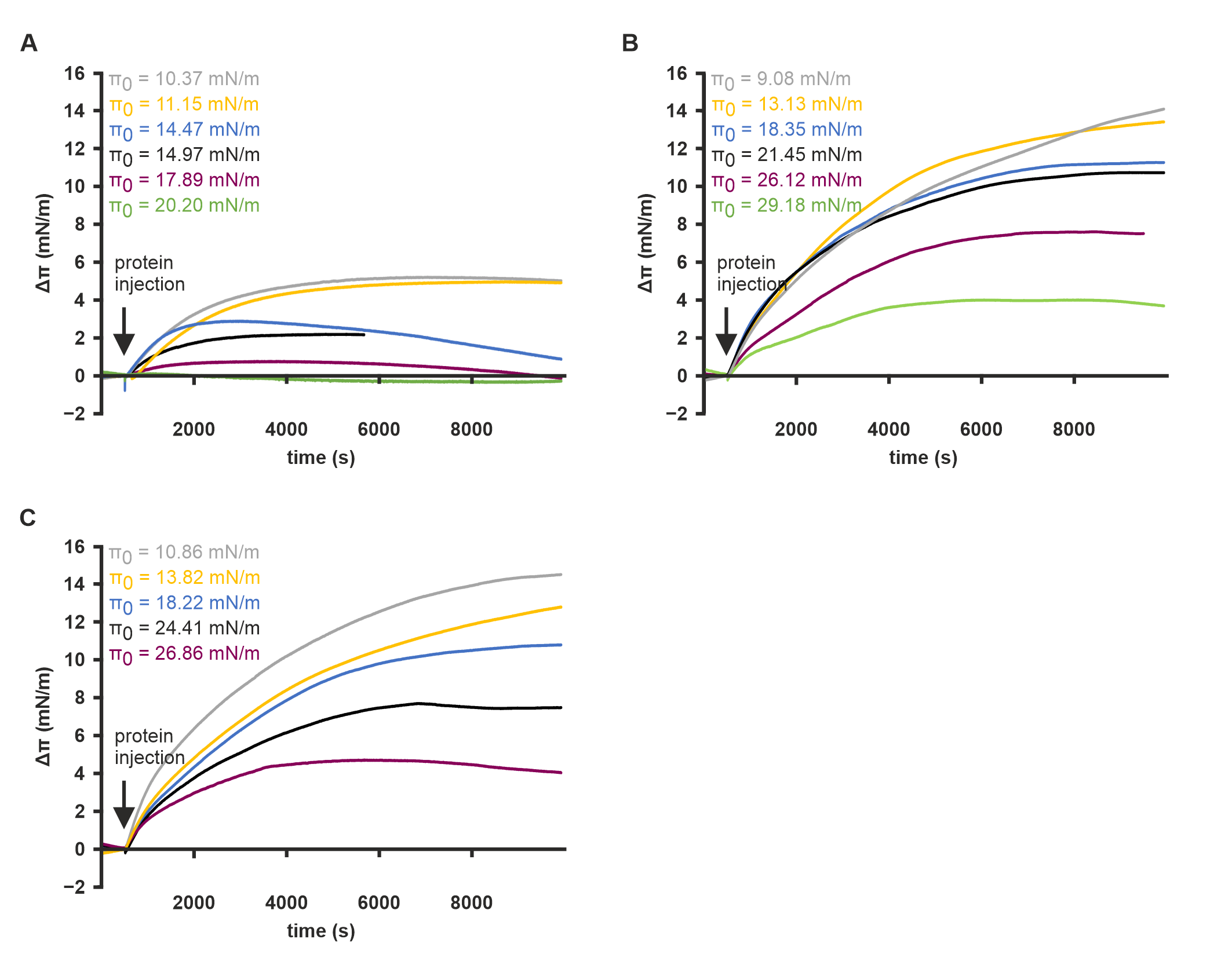

Supplement: S5 Fig — A Δπ curves for C protein injected with POPC monolayers. B Δπ curves for C protein injected with POPC/POPS monolayers. C Δπ curves for C18–93 protein injected with POPC/POPS monolayers. Data information: The π is zeroed to the preinjection pressure. The π0 values for each curve are indicated. (TIF) [file ppat.1011125.s005.tif]

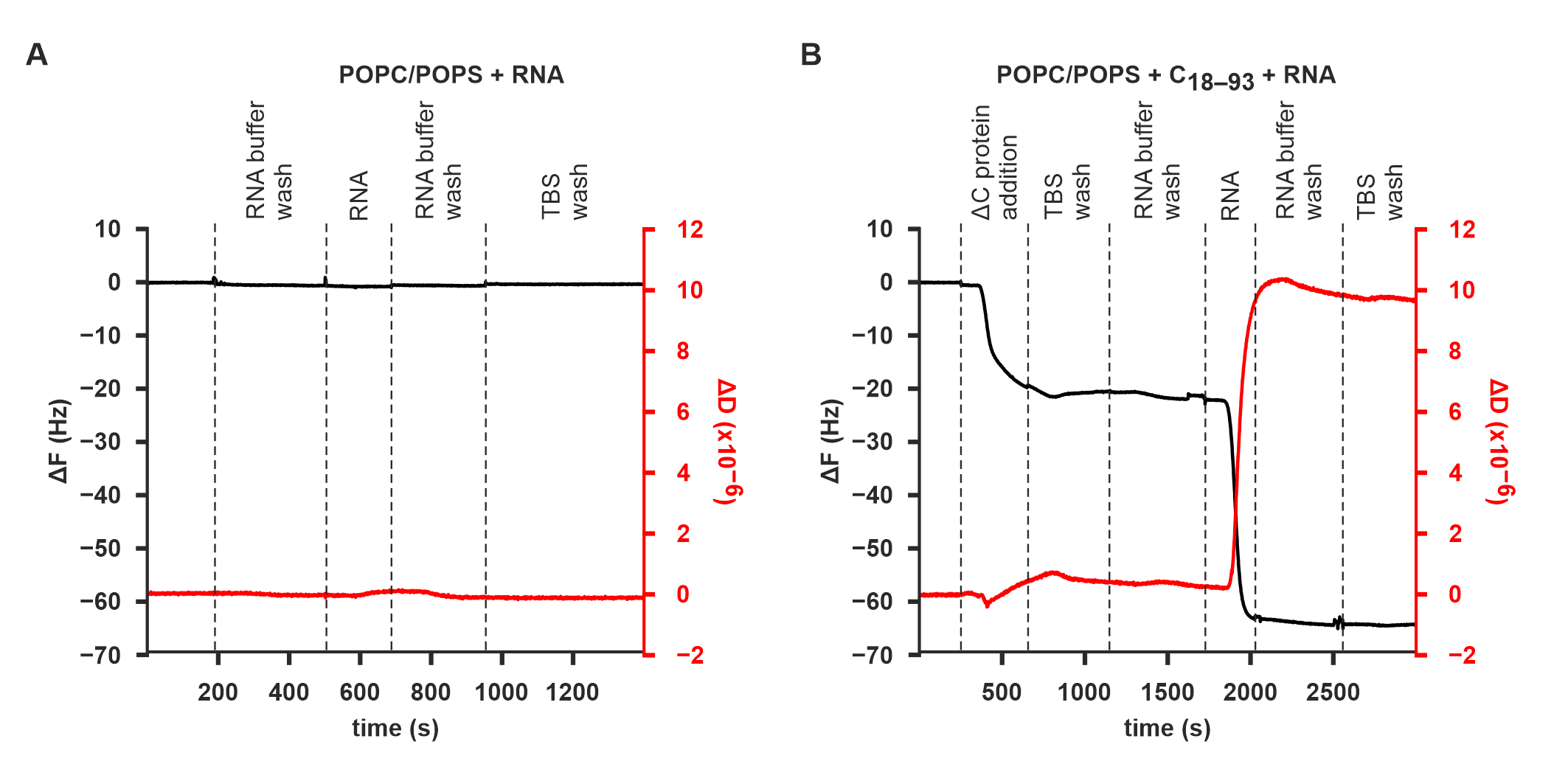

Supplement: S6 Fig — A Representative QCM-D curves from an RNA binding without protein pretreatment experiment on POPC/POPS SLBs. B Representative QCM-D curves from an RNA binding with pretreatment with C18–93 experiment on POPC/POPS SLBs. Data information: In each panel, the ΔF and ΔD have been zeroed to equilibrium values after SLB formation. (TIF) [file ppat.1011125.s006.tif]
