# Supplementary material for: Simultaneous membrane and RNA binding by tick-borne encephalitis virus capsid protein
Source: PLoS Pathog. 2023 Feb 14;19(2):e1011125. doi: 10.1371/journal.ppat.1011125 (PMC9970071; doi:10.1371/journal.ppat.1011125)
Supplement: Attachment — Submitted filename: full revision response to reviewers.pdf [file ppat.1011125.s008.pdf]

Full Revision

Manuscript number: RC- RC-2022-01707
Corresponding author(s): Sarah Butcher, Richard Lundmark

[Please use this template only if the submitted manuscript should be considered by the affiliate journal as a full revision in response to the points raised by the reviewers.

If you wish to submit a preliminary revision with a revision plan, please use our "Revision Plan" template. ***It is important to use the appropriate template to clearly inform the editors of your intentions.****]*

1. General Statements [optional]

This section is optional. Insert here any general statements you wish to make about the goal of the study or about the reviews.

We thank the reviewers for their insightful comments. The inclusion of the points raised by the referees have strengthened the manuscript. However, some of the reviewer suggestions are beyond the scope of the work (see below), but will doubtlessly be touched upon in future studies by the authors. In addition to incorporating changes relevant to answering the reviewers' comments, we have edited the manuscript for increased clarity and precision.

2. Point-by-point description of the revisions

This section is mandatory. Please insert a point-by-point reply describing the revisions that were already carried out and included in the transferred manuscript.

1. Reviewer #1
    a. "*One of the fundamental challenges of the work with flaviviral capsid proteins is that they tend to form amorphous aggregates to neutralize their highly positive surface charge. As the authors state themselves, „ We cannot rule out that the C protein preparation is heterogeneous..." I would strongly recommend supplementing the current liposome sedimentation assay by liposome flotation assay. In contrast to liposome co-sedimentation, the flotation assay can discriminate protein aggregates from proteins bound to liposomes. Although the SDS PAGE shown in Fig. 1A looks pretty convincing, a faint protein band in the „P" lane of the middle panel for the (-) sample is evident. Therefore, C protein aggregation cannot be ruled out and it would be indistinguishable from liposome binding examined by mere co-sedimentation assay. In addition, it needs to be clarified which TBEV C protein construct, whether full-length or truncated, was used for co-sedimentation fragmentation.*"

Response: We carried out the flotation assay using the full-length C protein and either DOPC/DOPE/rhodamine-DHPE/POPS (15:14:1:40 %, w/w) or DOPC/DOPE/rhodamine-DHPE (60:39:1 %, w/w). We have added an additional Supplementary figure showing a representative SDS-PAGE gel, and quantitation of three replicate experiments (Figure S3 inserted at line 618). In this figure, it can clearly be seen that the C-protein is highly enriched in the liposome fraction (2) when POPS is included, hardly any protein remains in the densest sucrose fraction, indicating that the protein is not aggregated or precipitated. We also added a paragraph in the Materials and Methods starting at line 485, as well as one in the Results starting at line 152.
We have clarified that the full-length C protein construct was used for the liposome co-sedimentation assays by adding "full-length" prior to instances of "C protein" e.g. in the paragraph starting line 118.

b. "*How to understand the finding that „the C protein forms a very rigid layer when adsorbed to the membrane". Can the aggregation of C-protein be ruled-out? Following the 1M NaCl wash of C-protein-bound to SLB, the authors stated: „This shows that initial membrane recruitment of C protein is strongly dependent on its interactions with the negatively-charged lipid headgroups. However, once bound, the C protein-membrane interaction is complemented with non-electrostatic interactions such as membrane insertion or protein oligomerization": does it mean that there are several layers of C protein, the first held by electrostatic interactions, overlayed by non-electrostatically bound C protein? If yes, the illustration of single-layered C-protein adsorbed onto SLB in Fig. 2A, B is not correct.* "

Response: We understand the confusion regarding the term "rigid" which was used as a way to describe how we interpret the relatively minor change in the dissipation upon membrane binding. What we intended to describe was that this indicates that the protein is attached in a stable way that does not add viscoelastic properties to the system. These data indicate that the protein does not form large aggregates that non-specifically attach to the membrane in different protrusive orientations. We have clarified this in the manuscript and specified that the as there is no dissipation change, there is no aggregation. We added the following to line 177 "This, in turn, indicates that the C protein does not bind as non-specific aggregates as these would have changed the viscoelastic properties of the system."

We do not mean that there are several layers of C protein. We consider, due to the highly charged nature of C, that the most likely explanation is that there are multiple modes of C binding but the result is only one layer, with multiple C-proteins interacting with each other within that layer. We have modified the text at line 193 to: "However, once bound, the C protein-membrane interaction is

complemented with non-electrostatic interactions such as membrane insertion or protein oligomerization within the bound layer."

c. "*The sentence: "To confirm that the C protein is biologically active, we investigated its ability to bind RNA" seems to be a little odd because it suggests the model membrane binding assays do not require biological active proteins. However, considering that the interactions leading to binding either negatively-charged lipid or negatively-charged RNA are electrostatic - this sentence must be rewritten."*

   Response: We thank the reviewer and have now rephrased this sentence to the following at line 258 "Since RNA binding is crucial for the NC assembly, we investigated the C protein's ability to perform this function."

d. *"The authors´ statement in the Abstract: „....we investigate nucleocapsid assembly..." is too speculative because the assembly was not studied in their work. It needs to be reformulated."*

   Response: We agree, and the statement has been removed from the abstract.

e. "*Despite this clear and valuable methodological contribution, the authors' contribution to our knowledge of the coordination of the nucleocapsid components to the sites of assembly and budding is not so obvious. Contrary to the earlier idea that the flavivirus is asymmetrically charged (that is, hydrophobic on one side (α2) and positively charged on the other side (α4), recent studies show that the entire surface of the protein is highly electropositive (Mebus-Antunnes et al., 2022). Therefore, a well-ordered neutralization of the flaviviral C proteins' highly positive surface seems critical for the proper organization and assembly of nucleocapsid. I am afraid that the authors do not shed much light on this issue*."

   Response: The recent structure of the TBEV C protein, published after we submitted the manuscript, shows that indeed the C protein is highly positively charged on all surfaces (updated Supplementary Figure 1 and Selinger et al., 2022). The recruitment of C protein to the membrane, that we demonstrate is dependent on negatively-charged head groups, provides a biologically relevant mechanism for charge neutralization on the C protein surface that interacts with

the lipids. The remaining surface charge can be then neutralized by RNA recruitment. Mebus-Antunnes et al. made their observations with just RNA and C protein from Dengue virus in the context of artificial surfaces e.g. mica. However, our experiments utilize the TBEV C protein and specifically include a membrane, the third critical component of NC assembly. Thus, we build upon the work of Mebus-Antunnes et al. by adding a second biologically relevant charge-neutralising component and comparing with a distantly-related virus. We have changed the discussion section of the manuscript to reflect this new structure and to emphasize the advance here. Starting from line 380 we changed the text to: "Recently, it has been shown that the neutralization of the C protein surface positive charge is important for RNA binding in the distantly-related Dengue virus (DENV) (Mebus-Antunes et al, 2022). The recruitment of C protein to the membrane, that we demonstrate is dependent on negatively-charged head groups, provides a biologically relevant mechanism for charge neutralization on the C protein surface that interacts with the lipids. The remaining surface charge can be then neutralized by RNA recruitment."

2. Reviewer #2
   a. "*What results demonstrate C protein inserts into membrane? The current results support the C protein interacts with membranes with positive charge, but do not seem to demonstrate membrane insertion. If the C protein inserts into the membrane, which regions (helices) play this role?*"

      Response: The Langmuir-Blodgett trough tensiometry experiments with monolayers directly measure the insertion of a protein into the monolayer. By determining the maximum insertion pressure of the C protein constructs, we also show that the membrane insertion can occur in bilayers. We show that the N-terminus is not inserting into the membrane, further work, beyond the scope of this manuscript, is needed to pinpoint the residues responsible for insertion, for instance by hydrogen-deuterium exchange or FRET measurements that would not affect folding. To clarify the use of the LB trough, we added the following at

line 225: “To investigate if the C protein membrane binding includes insertion into the membrane after the initial electrostatic binding, we used Langmuir-Blodgett trough monolayer experiments. In this approach, the insertion of a protein into a lipid monolayer can be detected by following the pressure (π) of the monolayer after protein injection into the aqueous subphase, with increases in π corresponding to protein injection (Brockman, 1999; Liu et al, 2022).“

b. “*The authors should discuss several previous papers reporting the effect of partial deletions of the C gene on the replication of TBEV, West Nile virus, and other flaviviruses.”*

Response: We agree that this is a necessary addition, and have now added a paragraph in the discussion section starting at line 343: “N-terminally truncated flaviviral C proteins have been shown to be assembly competent and in vitro, able to bind RNA, which is consistent with our results with N-terminally truncated TBEV C protein (Khromykh & Westaway, 1996; Kofler et al, 2002; Patkar et al, 2007; Schlick et al, 2009). Instead, a role for the N-terminus may be in the reported modulation of host responses to infection (Yang et al, 2002; Limjindaporn et al, 2007; Colpitts et al, 2011; Bhuvanakantham & Ng, 2013; Katoh et al, 2013; Urbanowski & Hobman, 2013; Samuel et al, 2016; Slomnicki et al, 2017; Fontaine et al, 2018). The membrane insertion directly detected in our experiments is central to C protein function. Other studies have found that deletions in the hydrophobic region of the α2 helix significantly impair particle assembly (Kofler et al, 2002; Patkar et al, 2007; Schlick et al, 2009). In the light of this evidence, we consider that the α2 helix could be responsible for membrane insertion (Markoff et al, 1997; Kofler et al, 2002; Nemésio et al, 2011, 2013).”

3. Reviewer #3
   a. “*In Figure 4, the band (256:1) that are supposedly in the wells (red arrow) is not clear- it is only slightly darker than the other wells.”*

   Response: This confusion was the result of unclear wording. We have now revised the figure legend at line 287 to : “The black arrow indicates the bands of freely-migrating RNA, and the red arrow the wells. On lanes 624:1 and 256:1, RNA has been immobilized in the wells.”

   b. “*Figure S1A, the N-terminal end (which is truncated in the mutant) should be colored on the cyan molecule.*”

   Response: We have coloured the truncated part of the cyan molecule in the figure (now S1B) according to the reviewer’s comment.

4. Other
    a. As the nuclear magnetic resonance structure of the truncated TBEV C protein has recently been released (Selinger et al, 2022), we have updated the manuscript and Figure S1 to include the information from this structure. We have also generated a new homology model of the full-length TBEV C protein using this structure as a template and included that in Figure S1.
